# Consistent zincophosphite 4-ring ‘ladder’ chain structural motif with isomeric ligands

**DOI:** 10.1107/S2056989023002062

**Published:** 2023-03-10

**Authors:** Stephen Wark, Megan J. Lyons, Alexandra M. Z. Slawin, William T. A. Harrison

**Affiliations:** a Department of Chemistry, University of Aberdeen, Meston Walk, Aberdeen AB24 3UE, Scotland, United Kingdom; b School of Chemistry, University of St Andrews, St Andrews KY16 9ST, Scotland, United Kingdom; University of Kentucky, USA

**Keywords:** crystal structure, zincophosphite, isomers

## Abstract

The syntheses and crystal structures of four hydro­thermally prepared organo–zinc phosphites, *viz*. poly[[(2-amino-3-methyl­pyridine)-μ_3_-phospho­nato-zinc] hemihydrate], {[Zn(HPO_3_)(C_6_H_8_N_2_)]·0.5H_2_O}_*n*_, (I), poly[(2-amino-4-methyl­pyridine)-μ_3_-phospho­nato-zinc], [Zn(HPO_3_)(C_6_H_8_N_2_)]_*n*_, (II), poly[(2-amino-5-methyl­pyridine)-μ_3_-phospho­nato-zinc], [Zn(HPO_3_)(C_6_H_8_N_2_)]_*n*_, (III), and poly[bis­(2-amino-4-methyl­pyridinium) [tetra-μ_3_-phospho­nato-trizinc] monohydrate], {(C_6_H_9_N_2_)_2_[Zn_3_(HPO_3_)_4_]·H_2_O}_*n*_, (IV), are described. Compounds (I)–(III) are constructed from vertex-sharing ZnO_3_N tetra­hedra (the organic mol­ecule acting as a ligand) and HPO_3_ pseudo pyramids in a 1:1 ratio to generate the same *motif* of infinite 4-ring ‘ladder’ chains propagating in the [010], [101] and [100] directions, respectively, whereas (IV) consists of (010) layers of vertex-sharing ZnO_4_ and HPO_3_ units in a 3:4 ratio with the protonated organic mol­ecule acting as a template. When an excess of HCl is used in the synthesis, the simple hydrated mol­ecular salt, bis­(2-amino-3-methyl­pyridinium) tetra­chloro­zincate monohydrate, (C_6_H_8_N_2_)_2_[ZnCl_4_]·H_2_O, (V), arises. Com­pounds (I)–(V) feature extensive networks of hydrogen bonds, both classical (N—H⋯O, N—H⋯Cl, O—H⋯O) and non-classical (C—H⋯O, C—H⋯Cl) in nature, which help to consolidate the extended structures.

## Abstract 2

The same ‘ladder’ chain motif built up from ZnO_3_N and HPO_3_ units arises for zincophosphites templated by isomers of 2-amino-*z*-methyl­pyridine (*z* = 3, 4, 5).

## 1. Chemical context

Since the first report (Harrison *et al.*, 2001 ▸) of zincophosphite (ZnPO) networks built up from vertex-sharing ZnO_4_ or ZnO_3_N and HPO_3_ building units templated or ligated by organic species, this family has grown to include well over 200 structures in the Cambridge Structural Database (CSD; Groom *et al.*, 2016 ▸). Recent papers have described a ZnPO templated by a chiral amino acid, which displays non-linear optical behaviour (Mao *et al.*, 2021 ▸) and a mixed-ligand ZnPO with promising gas sorption properties (Chen *et al.*, 2022 ▸). As well as their potential applications, ZnPOs are of ongoing academic inter­est in terms of the challenge of designing rational and reproducible syntheses and the elucidation of the systematics of their crystal chemistry, for example, the effect of the Zn:P ratio, different polyhedral connectivities, hydrogen bonding and the ‘dual role’ (bonded ligand or protonated guest) of the organic template on the structure (Holmes *et al.*, 2018 ▸).

In a continuation of our ongoing studies of these systems (Katinaitė & Harrison, 2017 ▸; Holmes *et al.*, 2018 ▸), we now describe the hydro­thermal syntheses and crystal structures of four organo–zinc phosphites featuring isomeric ligands, *viz*.: poly[[(2-amino-3-methyl­pyridine)-μ_3_-phospho­nato-zinc] hemihydrate], {[Zn(HPO_3_)(C_6_H_8_N_2_)]·0.5H_2_O}_*n*_, (I), poly[(2-amino-4-methyl­pyridine)-μ_3_-phospho­nato-zinc], [Zn(HPO_3_)(C_6_H_8_N_2_)]_*n*_, (II), poly[(2-amino-5-methyl­pyridine)-μ_3_-phos­pho­nato-zinc], [Zn(HPO_3_)(C_6_H_8_N_2_)]_*n*_, (III), and poly[bis­(2-amino-4-methyl­pyridinium) [tetra-μ_3_-phospho­nato-trizinc] monohydrate], {(C_6_H_9_N_2_)_2_[Zn_3_(HPO_3_)_4_]·H_2_O}_*n*_, (IV). The simple mol­ecular salt bis­(2-amino-3-methyl­pyridinium) tetra­chloro­zincate monohydrate, (C_6_H_8_N_2_)_2_·ZnCl_4_·H_2_O (V), is also described. 
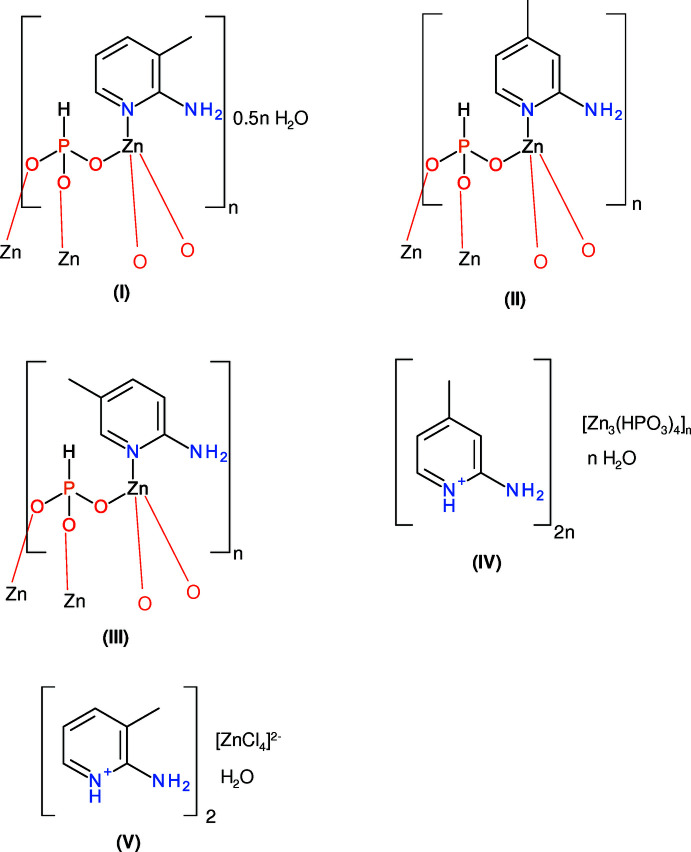


## 2. Structural commentary

**Figure 1:**
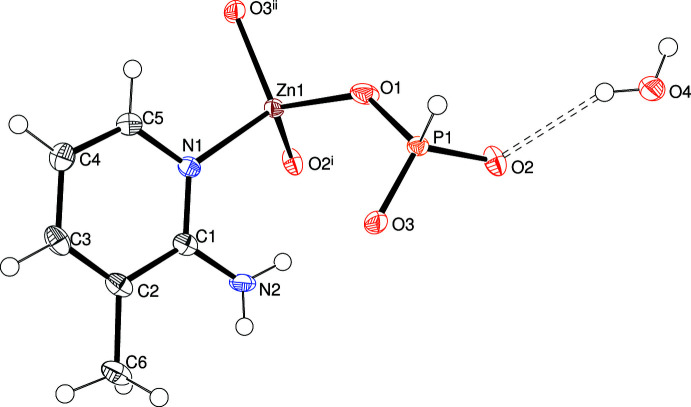
The asymmetric unit of (I) expanded to show the complete zinc-atom coordination sphere showing 50% displacement ellipsoids. Symmetry codes: (i) 1 − *x*, 1 − *y*, 1 − *z*; (ii) *x*, *y* + 1, *z*.

The asymmetric unit of (I) (Fig. 1 ▸), which crystallizes in the monoclinic space group *C*2/*c*, consists of a Zn^2+^ ion, a [HPO_3_]^2–^ hydrogen phosphite anion, a C_6_H_7_N_2_ 2-amino-3-methyl­pyridine mol­ecule and a water mol­ecule, the O atom of the last species lying on a crystallographic twofold axis. The zinc coordination polyhedron is a ZnO_3_N tetra­hedron, *i.e*., the organic species is acting as a ligand bonding to the metal ion from its pyridine nitro­gen atom and the Zn—O bonds (mean = 1.940 Å) are notably shorter than the Zn1—N1 [2.0262 (14) Å] link, as previously observed for related compounds (Holmes *et al.*, 2018 ▸). The spread of bond angles about the metal ion [minimum = 104.23 (5) for O2—Zn1—N1, maximum = 113.89 (6)° for O1—Zn1—N1] indicates a slight degree of distortion with τ_4_′ = 0.974 (Okuniewski *et al.*, 2015 ▸). The [HPO_3_]^2–^ group adopts its usual tetra­hedral (including the H atom) or pseudo-pyramidal (excluding H) geometry and the mean P—O separation is 1.522 Å with the O—P—O bond angles tightly clustered in the range 111.98 (7)–113.57 (7)°; the P atom is displaced by 0.4227 (8) Å from the plane of its attached O atoms. Each O atom is bonded to one Zn and one P atom [mean Zn—O—P = 130.2°], thus there are no ‘dangling’ (Holmes *et al.*, 2018 ▸) Zn—OH_2_, P=O or P—OH bonds in this structure. The extended structure of (I) is discussed below.

**Figure 2:**
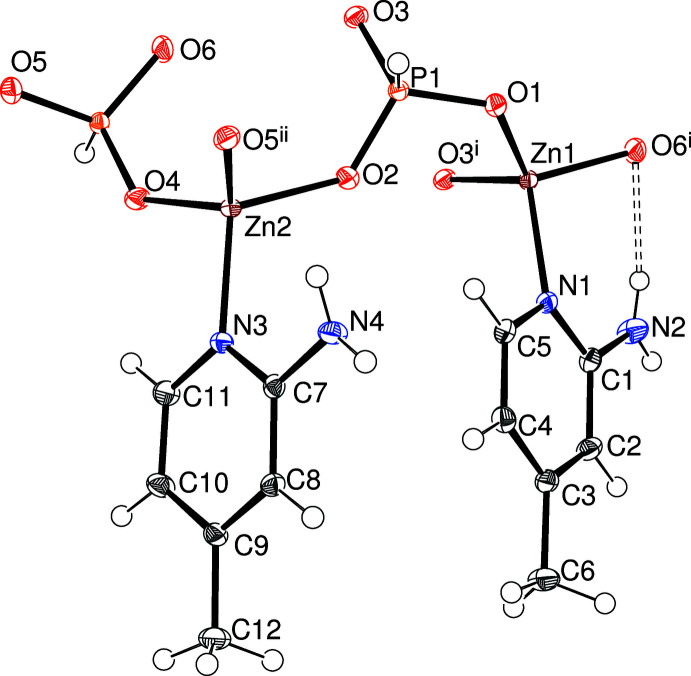
The asymmetric unit of (II) expanded to show the complete zinc-atom coordination spheres showing 50% displacement ellipsoids. Symmetry codes: (i) 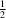 − *x*, 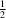 − *y*, 1 − *z*; (ii) 1 − *x*, *y*, 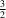 − *z*.

The asymmetric unit of (II) (Fig. 2 ▸), which also crystallizes in *C*2/*c*, consists of two Zn^2+^ ions, two [HPO_3_]^2–^ hydrogen phosphite anions, and two C_6_H_7_N_2_ 2-amino-4-methyl­pyridine mol­ecules acting as ligands, *i.e.*, *Z*′ = 2. Unlike (I), (II) does not contain any water mol­ecules of crystallization. The building units – vertex sharing ZnO_3_N tetra­hedra and [HPO_3_]^2–^ dianions – and the major structural features of (II) are broadly similar to those of (I): mean Zn1—O = 1.942 Å; spread of O—Zn1—O/O—Zn1—N bond angles = 104.06 (5)–114.29 (5)°, τ_4_′ = 0.97; comparable data for Zn2 = 1.940 Å, 100.10 (5)–121.28 (5)° and 0.91, respectively; mean P1—O = 1.525 Å; spread of O—P1—O bond angles = 110.64 (6)–113.78 (6)°; P1 displacement from its attached O atoms = 0.4278 (7) Å; comparable data for P2 = 1.520 Å, 111.44 (6)–113.14 (7)° and −0.4269 (8) Å, respectively. All six O atoms are bridging between Zn and P atoms with a mean bond angle of 131.3° [range = 123.26 (6)–144.09 (8)°]. The extended structure of (II) is discussed below.

**Figure 3:**
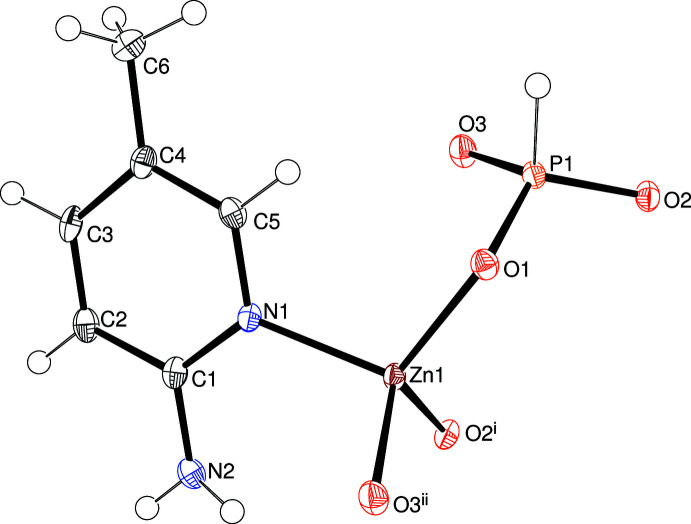
The asymmetric unit of (III) expanded to show the complete zinc-atom coordination sphere showing 50% displacement ellipsoids. Symmetry codes: (i) 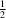 + *x*, 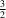 − *y*, −*z*; (ii) 1 + *x*, *y*, *z*.

Compound (III) crystallizes in the ortho­rhom­bic space group *P*2_1_2_1_2_1_ with a well-defined absolute structure and its asymmetric unit (Fig. 3 ▸) consists of a Zn^2+^ ion, a [HPO_3_]^2–^ hydrogen phosphite anion and a C_6_H_7_N_2_ 2-amino-5-methyl­pyridine mol­ecule bonded to the metal ion from its pyridine N atom. Once again, the constituent polyhedra are ZnO_3_N tetra­hedra [mean Zn—O = 1.941 Å, minimum and maximum bond angles = 105.19 (9) and 115.09 (8)°, respectively, τ_4_′ = 0.96] and [HPO_3_]^2–^ units [mean P—O = 1.522 Å, minimum and maximum O—P—O = 110.43 (11) and 113.86 (12)°, respectively, deviation of P1 from O1/O2/O3 = 0.4237 (14) Å]. The three O atoms bridge adjacent zinc and phospho­rus atoms with a mean Zn—O—P bond angle of 126.7°. For the extended structure of (III), see below.

**Figure 4:**
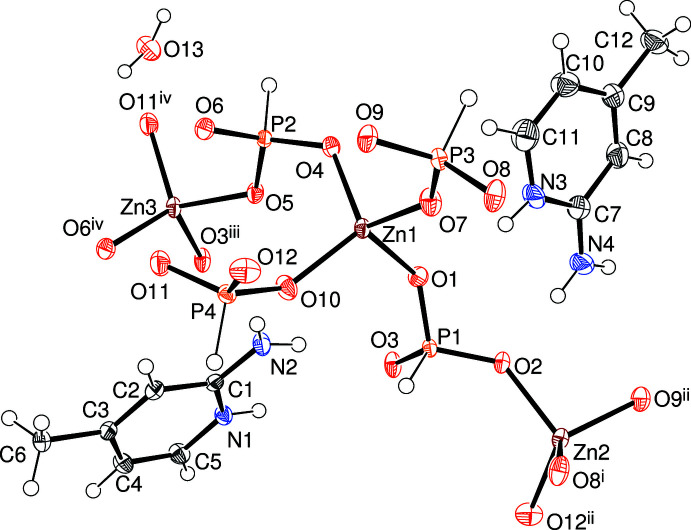
The asymmetric unit of (IV) expanded to show the complete zinc-atom coordination sphere showing 50% displacement ellipsoids. Symmetry codes: (i) −*x*, 1 − *y*, 1 − *z*; (ii) *x* − 1, *y*, *z*; (iii) −*x*, 1 − *y*, −*z*; (iv) 1 − *x*, 1 − *y*, −*z*.

In (IV), which crystallizes in the triclinic space group *P* 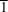 , the expanded asymmetric unit (Fig. 4 ▸) reveals different constit­uent polyhedra of three distinct ZnO_4_ tetra­hedra and four [HPO_3_^2–^] pseudo pyramids as well as two protonated 2-amino-4-methyl­pyridinium cations, which therefore act as templates rather than ligands; a water mol­ecule of crystallization (O13) completes the structure. Geometrical data for the zinc polyhedra are as follows: mean Zn1—O = 1.941 Å, spread of bond angles = 100.42 (8)–122.18 (9)°, τ_4_’ = 0.90; equivalent data for Zn2: 1.936 Å, 98.33 (8)–115.30 (9)° and 0.98, respectively; equivalent data for Zn3: 1.945 Å, 99.70 (8)–117.10 (8)° and 0.96, respectively. The four [HPO_3_]^2–^ anions adopt their normal geometries: mean P1—O = 1.519 Å, minimum and maximum O—P1—O = 111.00 (11) and 112.70 (11)°, respectively, deviation of P1 from its attached O atoms = 0.4498 (13) Å; equivalent data for P2: 1.522 Å, 110.08 (10)°, 115.33 (11)° and −0.4122 (12) Å, respectively; equivalent data for P3: 1.516 Å, 110.19 (11)°, 114.49 (12)° and −0.4123 (13) Å, respectively; equivalent data for P4: 1.516 Å, 112.68 (12)°, 114.13 (12)° and 0.3903 (13) Å, respectively. The twelve unique O atoms all bridge Zn and P atoms (mean bond angle = 134.9, minimum = 125.40 (11), maximum = 146.90 (13), spread = 21.5°). For the extended structure of (IV), see below.

**Figure 5:**
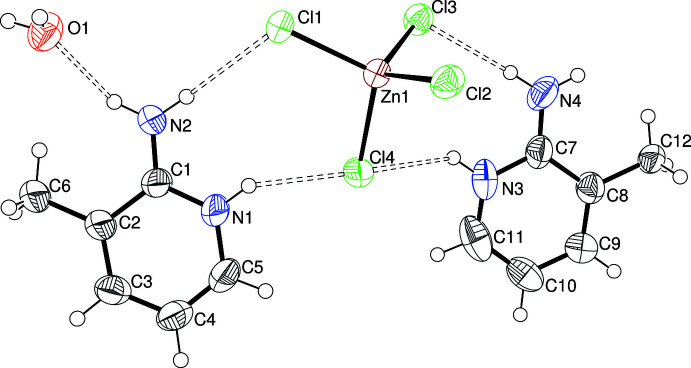
The asymmetric unit of (V) showing 50% displacement ellipsoids. Hydrogen bonds are indicated by double-dashed lines.

Compound (V) is a simple mol­ecular salt (Fig. 5 ▸), which crystallizes in the triclinic space group *P* 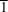 : its asymmetric unit consists of two 2-amino-3-methyl­pyridinium C_6_H_8_N_2_^+^ cations protonated at their pyridine N atoms, a [ZnCl_4_]^2–^ anion and a water mol­ecule of crystallisation. The tetra­chloro­zincate ion has a mean Zn—Cl separation of 2.2704 Å [range = 2.2536 (13)–2.2867 (13) Å] and smallest and largest Cl—Zn—Cl bond angles of 104.48 (5) and 113.75 (5)°, respectively. The synthetic intent here was to lower the pH with HCl and establish if a di­hydrogen phosphite (H_2_PO_3_^−^) anion containing a terminal P—OH moiety could be incorporated into the structure (Lin *et al.*, 2003 ▸) but the presence of excess chloride ions has led to a completely different and unwanted mol­ecular salt containing the tetra­chloro­zincate complex ion, which has been reported many times before, with over 1000 matches in the CSD.

## 3. Supra­molecular features

**Figure 6:**
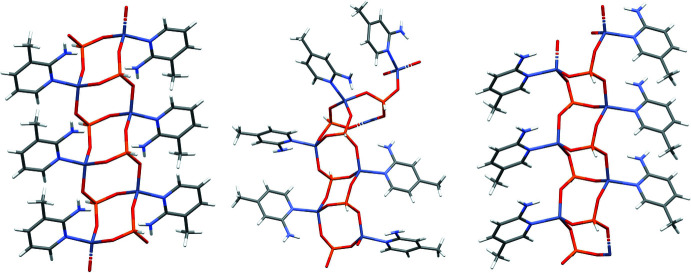
Comparison of the zincophosphite 4-ring ladder chains in the extended structures of (I) (left), (II) (centre) and (III) (right).

In the extended structure of (I), the constituent ZnO_3_N and HPO_3_ polyhedra are linked by Zn—O—P bonds into [010] polyhedral 4-ring (two Zn and two P nodes) ‘ladder’ chains in which the zinc and phospho­rus nodes strictly alternate (Fig. 6 ▸): the chains are built up by inversion symmetry at the centres of every 4-ring, as well as, of course, translation symmetry in the *b*-axis direction. Given that the Zn atom forms three bonds (*via* O atoms) to adjacent P atoms (and a fourth bond to the organic species) and that the P atom forms three links to zinc atoms (and a fourth P—H vertex), the 1:1 Zn:P stoichiometry is to be expected and hence no charge compensating, protonated template is needed. In (II), ladder chains similar to those seen in (I) arise in the extended structure (Fig. 6 ▸) although they are more contorted: because *Z*′ = 2, every other 4-ring is generated by inversion symmetry and translation in the [101] direction leads to the extended array. In (III), the 4-ring ladder *motif* is again apparent (Fig. 6 ▸), although in this case, the combination of a 2_1_ screw-axis parallel to the chain and *a*-translation symmetry generates the infinite [100] chains. In each structure, the organic mol­ecules are pendant to the chains (Fig. 6 ▸).

**Figure 7:**
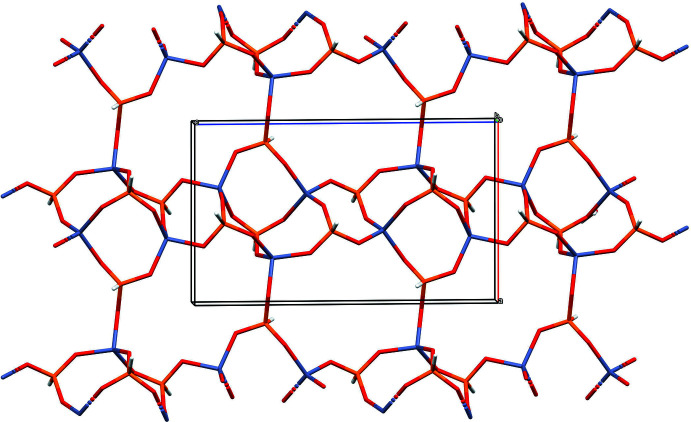
Part of an infinite (010) layer of vertex sharing ZnO_4_ and HPO_3_ moieties in the extended structure of (IV) viewed down [010].

The extended structure of (IV) (Fig. 7 ▸) is quite different to those of (I)–(III) and features (010) sheets of ZnO_4_ and HPO_3_ polyhedra sharing corners. One way to visualize this rather complex arrangement (although this does not necessarily imply that the synthesis proceeds in such a step-by-step fashion) is in terms of contorted chains of 4-rings featuring atoms Zn1, Zn2, Zn3, P2, P3 and P4 as the nodes propagating in the [001] direction. One out of every three 4-rings in a chain is generated by inversion symmetry. These chains are cross-linked in the *a*-axis direction by the P1-centred hydrogen phosphite groups to form the (010) layers, which encapsulate 8-ring voids built up from four Zn and four P nodes although there is no suggestion of ‘zeolitic’ porosity. So far as stoichiometry is concerned, in this case the zinc nodes forming four bonds (*via* all their O atoms) to nearby phospho­rus atoms and the P nodes forming three bonds to Zn atoms leads to the 3:4 ratio of zinc and phospho­rus, which is the proportion most commonly seen in this family of phases (*e.g*., Phillips *et al.*, 2002 ▸; Lin *et al.*, 2009 ▸). In this case, the inorganic component bears a charge of −2 per [Zn_3_(HPO_3_)_4_] unit, hence the two protonated template mol­ecules. The template cations and water mol­ecules of crystallisation occupy the inter-layer regions.

**Figure 8:**
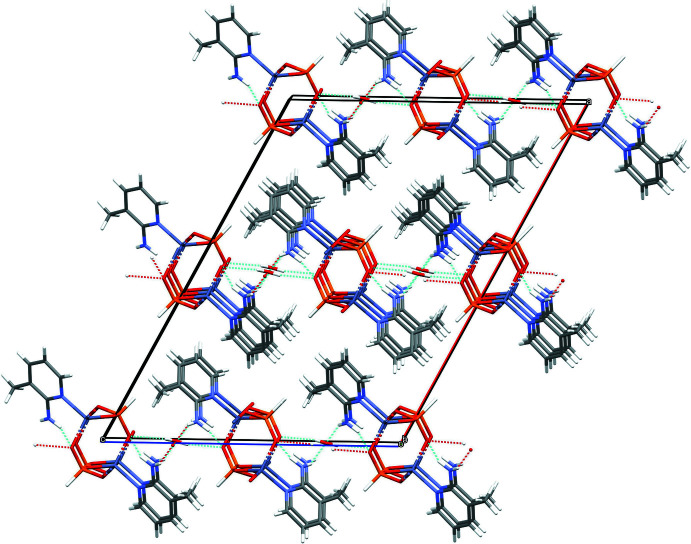
The unit-cell packing in (I) viewed down [001]. Hydrogen bonds are shown as dashed lines.

Various classical (N—H⋯O, N—H⋯Cl and O—H⋯O) and non-classical (C—H⋯O and C—H⋯Cl) hydrogen bonds occur in these structures. As is normal, the hydrogen phosphite P—H unit does not participate in hydrogen bonding (Katinaitė & Harrison, 2017 ▸). In (I), the water mol­ecule of crystallization, which lies on a crystallographic twofold axis, appears to play an important role in consolidating the extended structure by accepting two N—H⋯O hydrogen bonds (Table 1 ▸) and donating two O—H⋯O hydrogen bonds to cross-link the [001] chains into (100) layers (Fig. 8 ▸). The other hydrogen bond arising from the amine group is an intra-chain N—H⋯O link. There are no aromatic π–π stacking inter­actions in (I), the shortest centroid–centroid separation being some 5.04 Å.

**Figure 9:**
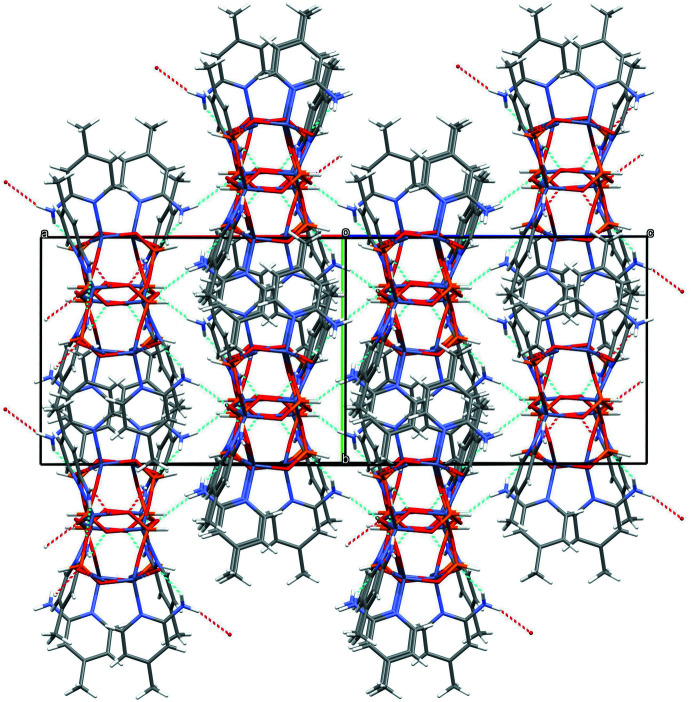
The unit-cell packing in (II) viewed down [101]. Hydrogen bonds are shown as dashed lines.

**Figure 10:**
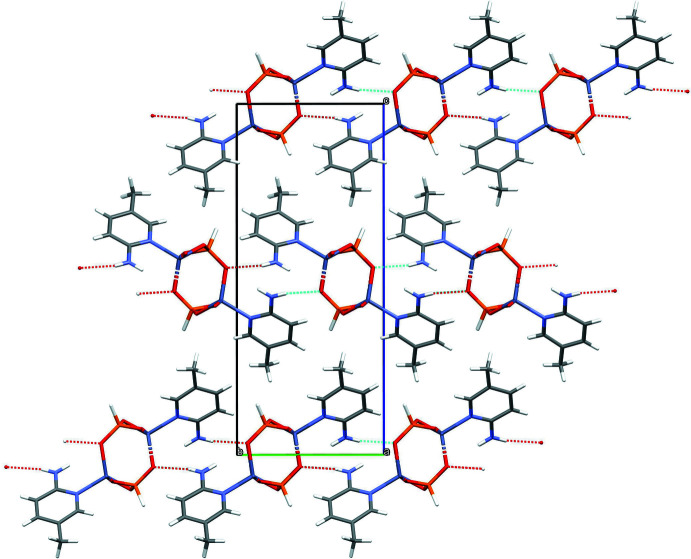
The unit-cell packing in (III) viewed down [100]. Hydrogen bonds are shown as dashed lines.

In (II), the N—H⋯O hydrogen bonds arising from the amine groups are a mix of intra- (*via* H2*N* and H4*N*) and inter-chain (*via* H1*N* and H3*N*) links, with the latter serving to cross-link the [101] chains into a three-dimensional network (Table 2 ▸; Fig. 9 ▸). The aromatic rings are inter­digitated and this is reflected in the shortest centroid–centroid separation of 3.8234 (17) Å. In the extended structure of (III) (Fig. 10 ▸), the single N1—H2*B*⋯O2 bond (Table 3 ▸) cross-links the [100] chains into (001) sheets. The other hydrogen atom (H2*A*) of the amine grouping does not participate in a hydrogen bond, the closest acceptor O atom being some 2.77 Å distant. There are no significant π–π stacking inter­actions in (III) [shortest centroid–centroid separation = 5.149 (2) Å].

**Figure 11:**
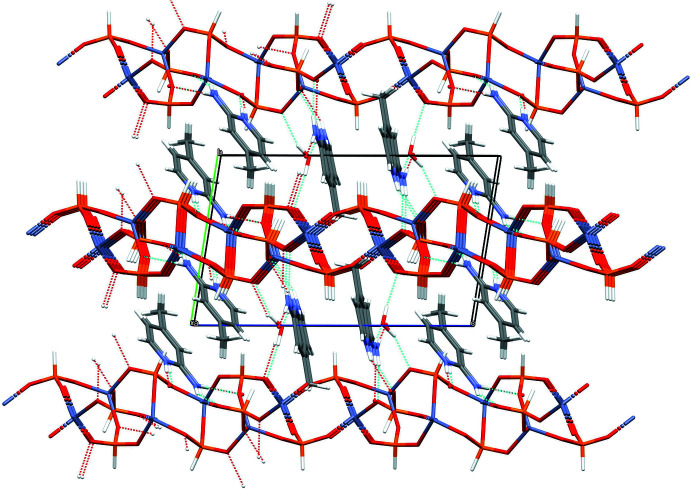
The unit-cell packing in (IV) viewed down [100]. Hydrogen bonds are shown as dashed lines.

In (IV), numerous hydrogen bonds are observed (Table 4 ▸, Fig. 11 ▸). The water mol­ecule cross-links adjacent (010) layers *via* two O—H⋯O hydrogen bonds. The N—H⋯O hydrogen bonds originating from the protonated pyridine N atoms and the –NH_2_ groups of the organic species all link to the same sheet for each template cation, *i.e*., there are no inter-sheet hydrogen bonds associated with the templates. Significant aromatic π–π stacking inter­actions occur between centrosymmetric pairs of each template cation, as indicated by the centroid–centroid separation of 3.6167 (15) Å (slippage = 1.196 Å) for the C1 species and 3.4695 (17) Å (0.146 Å) for the C7 cation.

**Figure 12:**
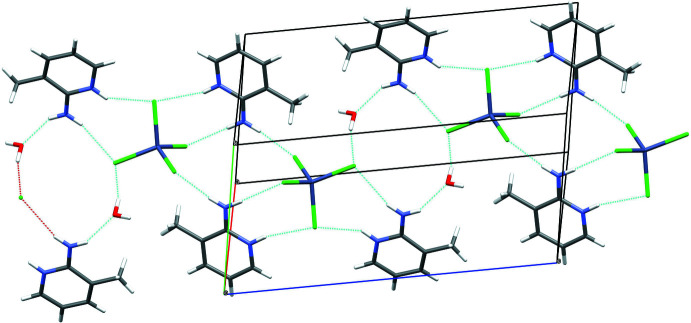
The unit-cell packing in (V) viewed approximately down [10 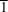 ]. Hydrogen bonds are shown as dashed lines.

In the extended structure of (V), the hydrogen-bond scheme is completely different (Table 5 ▸) and the component cations, anions and water mol­ecules are linked by N—H⋯Cl, N—H⋯O_w_ (w = water) and O—H⋯Cl inter­actions to generate [001] chains; within these chains, centrosymmetric assemblages of two C_6_H_8_N_2_^+^ cations, two [ZnCl_4_]^2–^ anions and two water mol­ecules are apparent (Fig. 12 ▸).

## 4. Database survey

A survey of the Cambridge Structural Database (Groom *et al.*, 2016 ▸; updated to February 2023) revealed 213 crystal structures containing zinc cations and hydrogenphosphite anions (Zn—O—P—H fragment) of which 53 contain a ligated organic mol­ecule (Zn—N bond). The only phase that bears a close chemical similarity to the structures described here is [C_5_H_6_N_2_·Zn(HPO_3_)]_*n*_, *catena*-[(μ_3_-hydrogenphosphito)(2-amino­pyridine)­zinc] (CSD refcode LUZYOU) (Liang *et al.*, 2003 ▸), in which the ZnO_3_N and HPO_3_ polyhedra assemble into (100) layers of 4- and 8-rings.

The fact that the N-bonded zinc ions and HPO_3_ units in (I), (II) and (III) self assemble to form the same 4-ring ladder chain with different isomeric pyridine-based ligands suggests that it is a reasonably robust structural feature. However, it is not a particularly common *motif* in the wider ZnPO phase space: two other examples with very different ligating mol­ecules to those in (I)–(III) are [C_4_H_8_N_2_O_3_·Zn(HPO_3_)]_*n*_ (C_4_H_8_N_2_O_3_ = l-asparagine) (Gordon & Harrison, 2004 ▸) and [C_3_H_7_NO_2_·Zn(HPO_3_)]_*n*_ (C_3_H_7_NO_2_ = racemic dl-alanine) (Mao *et al.*, 2021 ▸); it is notable that these amino acids both bond to the zinc atom *via* one of their carboxyl­ate O atoms rather than the pyridine N atoms in (I)–(III).

Compound (II), in which the C_6_H_8_N_2_ organic mol­ecule acts as a ligand (a Zn—N bond and a 1:1 Zn:P ratio) and (IV), in which the same organic species acts as a protonated C_6_H_9_N_2_^+^ template (N—H⋯O hydrogen bonds and a 3:4 Zn:P ratio) arose from similar syntheses, with the only difference being the source of zinc ions (zinc oxide and zinc acetate, respectively). Assuming that hydro­thermal synthesis is not just an impenetrable ‘black box’ (Ursu *et al.*, 2022 ▸), we may speculate that the acetate synthesis occurred at a lower pH, perhaps with some buffering action between acetic acid formed *in situ* and acetate ions, to allow for the protonation of the template.

## 5. Synthesis and crystallization

Compound (I) was prepared by mixing 0.77 g of ZnO, 0.76 g of H_3_PO_3_ and 1.14 g of 2-amino-3-methyl­pyridine (Zn:P:template ratio ≃ 1:1:1), which were placed in a 50 ml polypropyl­ene bottle with 20 ml of water and shaken well to result in a white slurry. The bottle was placed in an 353 K oven for 24 h and then removed and allowed to cool to room temperature. The solids were recovered by vacuum filtration to result in a mass of needle-like transparent crystals. IR: 2383 cm^−1^ (P—H stretch). Increasing the heating time to one week led to the same product, with a slight improvement in crystallinity, as indicated by sharper peaks in its IR spectrum and X-ray powder diffraction pattern.

Compound (II) was prepared from 0.75 g of ZnO, 0.81 g of H_3_PO_3_ and 1.10 g of 2-amino-4-methyl­pyridine (Zn:P:template ratio ≃ 1:1:1); otherwise following the same procedure as for (I). A mass of blocky transparent crystals was recovered. IR: 2394, 2382 cm^−1^ (P—H stretch). Two peaks may arise because of the two different P—H groups in the asymmetric unit (Ma *et al.*, 2007 ▸).

To prepare compound (III), 2.20 g of Zn(OAc)_2_, 0.86 g of H_3_PO_3_ and 1.09 g of 2-amino-5-methyl­pyridine (Zn:P:template ratio ≃ 1:1:1) and 20 ml of water were placed in a 50 ml polypropyl­ene bottle and heated to 353 K for three days. Upon cooling, the product consisted of a mass of colourless blocks. IR: 2406 cm^−1^ (P—H stretch).

Compound (IV) started from a mixture of 2.02 g Zn(OAc)_2_, 0.77 g of H_3_PO_3_ and 1.03 g of 2-amino-4-methyl­pyridine (Zn:P:template ratio ≃ 1:1:1) and 20 ml of water. These components were placed in a 50-ml polypropyl­ene bottle and heated to 353 K for 24 h. Upon cooling, the product consisted of a mass of colourless blocks. IR: 3000–3600 (broad) (O—H stretch), 2391, 2381 cm^−1^ (P—H stretch). The same product arises if the mixture is heated for one week.

Compound (V) was prepared from the same reagents as (I) and the same synthesis procedure but with the addition of 10 ml of 1 *M* HCl.

## 6. Refinement

Crystal data, data collection and structure refinement details are summarized in Table 6 ▸. Most of the O- and N-bound H atoms were located in difference maps and their positions were freely refined with *U*_iso_(H) = 1.2*U*_eq_(N or O). The phosphite H atoms were geometrically placed (P—H = 1.32 Å) and refined as riding atoms with *U*_iso_(H) = 1.2*U*_eq_(P). All the C-bound H atoms were located geometrically (C—H = 0.95–0.98 Å) and refined as riding atoms with *U*_iso_(H) = 1.2*U*_eq_(C) or 1.5*U*_eq_(methyl C). The methyl groups were allowed to rotate, but not to tip, to best fit the electron density. Two peaks greater than 1 e Å^−3^ were found in the final difference map for (IV) in the vicinity of the C7 cation but they did not correspond to a plausible chemical feature. The data quality for (V) was notably poorer than for the other four crystals.

## Supplementary Material

Crystal structure: contains datablock(s) I, II, III, IV, V, global. DOI: 10.1107/S2056989023002062/pk2681sup1.cif

Structure factors: contains datablock(s) I. DOI: 10.1107/S2056989023002062/pk2681Isup2.hkl

Structure factors: contains datablock(s) II. DOI: 10.1107/S2056989023002062/pk2681IIsup3.hkl

Structure factors: contains datablock(s) III. DOI: 10.1107/S2056989023002062/pk2681IIIsup4.hkl

Structure factors: contains datablock(s) IV. DOI: 10.1107/S2056989023002062/pk2681IVsup5.hkl

Structure factors: contains datablock(s) V. DOI: 10.1107/S2056989023002062/pk2681Vsup6.hkl

CCDC references: 2246111, 2246110, 2246109, 2246108, 2246107

Additional supporting information:  crystallographic information; 3D view; checkCIF report

## Figures and Tables

**Table 1:** Hydrogen-bond geometry (Å, °) for (I)

*D*—H⋯*A*	*D*—H	H⋯*A*	*D*⋯*A*	*D*—H⋯*A*
N2—H1*N*⋯O4^i^	0.78 (2)	2.19 (2)	2.919 (2)	157 (2)
N2—H2*N*⋯O2^ii^	0.84 (2)	2.54 (2)	3.098 (2)	124.8 (18)
O4—H1*O*⋯O2	0.79 (2)	2.199 (19)	2.9165 (15)	152 (2)

Symmetry codes: (i) 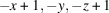 ; (ii) 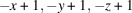 .

**Table 2:** Hydrogen-bond geometry (Å, °) for (II)

*D*—H⋯*A*	*D*—H	H⋯*A*	*D*⋯*A*	*D*—H⋯*A*
N2—H1*N*⋯O3^i^	0.79 (2)	2.35 (2)	2.9056 (19)	128 (2)
N2—H2*N*⋯O6^ii^	0.82 (2)	2.13 (2)	2.900 (2)	155 (2)
N4—H3*N*⋯O1^iii^	0.88 (2)	2.18 (2)	3.0310 (18)	163.7 (18)
N4—H4*N*⋯O5^iv^	0.83 (2)	2.39 (2)	3.1555 (19)	154.3 (18)
C5—H5⋯O2	0.95	2.57	3.315 (2)	136
C8—H8⋯O1^iii^	0.95	2.65	3.405 (2)	137
C11—H11⋯O4	0.95	2.51	3.105 (2)	120

Symmetry codes: (i) 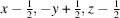 ; (ii) 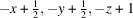 ; (iii) 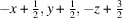 ; (iv) 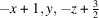 .

**Table 3:** Hydrogen-bond geometry (Å, °) for (III)

*D*—H⋯*A*	*D*—H	H⋯*A*	*D*⋯*A*	*D*—H⋯*A*
N2—H2*B*⋯O2^i^	0.79 (4)	2.35 (4)	3.111 (3)	162 (3)
C2—H2⋯O1^ii^	0.95	2.55	3.212 (3)	127

Symmetry codes: (i) 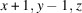 ; (ii) 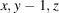 .

**Table 4:** Hydrogen-bond geometry (Å, °) for (IV)

*D*—H⋯*A*	*D*—H	H⋯*A*	*D*⋯*A*	*D*—H⋯*A*
N1—H1*A*⋯O5^i^	0.88	1.97	2.833 (3)	166
N2—H2*N*⋯O3	0.85 (4)	2.05 (4)	2.853 (3)	157 (3)
N2—H3*N*⋯O10	0.85 (4)	2.16 (4)	2.961 (3)	156 (3)
N3—H4*N*⋯O1	0.83 (4)	2.07 (4)	2.873 (3)	163 (4)
N4—H5*N*⋯O13^ii^	0.93 (4)	1.95 (4)	2.871 (4)	177 (4)
N4—H6*N*⋯O2	0.88 (4)	2.05 (4)	2.918 (3)	167 (4)
O13—H1*O*⋯O12^iii^	0.98	2.05	3.028 (3)	175
O13—H2*O*⋯O4^iv^	0.96	2.05	2.952 (3)	157
C5—H5⋯O4^i^	0.95	2.64	3.370 (3)	134
C8—H8⋯O8^v^	0.95	2.48	3.350 (4)	152
C11—H11⋯O7	0.95	2.47	3.199 (4)	133
C11—H11⋯O13^iv^	0.95	2.59	3.281 (4)	130

Symmetry codes: (i) 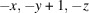 ; (ii) 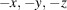 ; (iii) 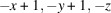 ; (iv) 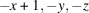 ; (v) 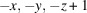 .

**Table 5:** Hydrogen-bond geometry (Å, °) for (V)

*D*—H⋯*A*	*D*—H	H⋯*A*	*D*⋯*A*	*D*—H⋯*A*
N1—H1⋯Cl4	0.88	2.35	3.134 (5)	148
N2—H2*A*⋯Cl1	0.88	2.54	3.375 (4)	158
N2—H2*B*⋯O1	0.88	2.03	2.838 (5)	152
N3—H3*A*⋯Cl4	0.88	2.66	3.306 (4)	132
N4—H4*A*⋯Cl3	0.88	2.40	3.268 (4)	170
N4—H4*B*⋯Cl2^i^	0.88	2.51	3.333 (5)	155
O1—H1*O*⋯Cl3^ii^	0.88	2.95	3.669 (4)	141
O1—H1*O*⋯Cl4^ii^	0.88	2.98	3.675 (4)	138
O1—H2*O*⋯Cl1^iii^	0.88	2.45	3.302 (4)	162
C10—H10⋯Cl3^iv^	0.95	2.92	3.696 (5)	140

Symmetry codes: (i) 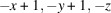 ; (ii) 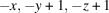 ; (iii) 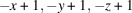 ; (iv) 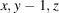 .

**Table 6:** Experimental details

	(I)	(II)	(III)	(IV)	(V)
Crystal data					
Chemical formula	[Zn(HPO_3_)(C_6_H_8_N_2_)]·0.5H_2_O	[Zn(HPO_3_)(C_6_H_8_N_2_)]	[Zn(HPO_3_)(C_6_H_8_N_2_)]	(C_6_H_9_N_2_)_2_[Zn_3_(HPO_3_)_4_]·H_2_O	(C_6_H_8_N_2_)_2_[ZnCl_4_]·H_2_O
*M*_r_	525.00	253.49	253.49	752.34	443.49
Crystal system, space group	Monoclinic, *C*2/*c*	Monoclinic, *C*2/*c*	Orthorhombic, *P*2_1_2_1_2_1_	Triclinic, *P* 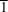	Triclinic, *P* 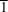
Temperature (K)	93	93	93	93	173
*a*, *b*, *c* (Å)	23.282 (6), 5.1926 (1), 17.738 (5)	22.873 (6), 12.307 (3), 16.633 (4)	5.1487 (9), 8.5316 (19), 20.371 (5)	9.03805 (13), 9.36837 (13), 15.0207 (2)	6.9541 (8), 8.7092 (9), 16.293 (3)
α, β, γ (°)	90, 117.974 (4), 90	90, 128.954 (5), 90	90, 90, 90	81.0313 (1), 88.0646 (1), 80.0782 (1)	83.239 (11), 80.167 (10), 72.049 (9)
*V* (Å^3^)	1893.9 (7)	3641.1 (16)	894.8 (3)	1237.46 (3)	922.7 (2)
*Z*	4	16	4	2	2
Radiation type	Mo *K*α	Mo *K*α	Mo *K*α	Cu *K*α	Mo *K*α
μ (mm^−1^)	2.75	2.85	2.90	6.49	1.92
Crystal size (mm)	0.20 × 0.02 × 0.02	0.10 × 0.03 × 0.03	0.15 × 0.05 × 0.03	0.10 × 0.10 × 0.10	0.15 × 0.05 × 0.05

Data collection					
Diffractometer	Rigaku Pilatus 200K CCD	Rigaku Pilatus 200K CCD	Rigaku Pilatus 200K CCD	Rigaku Pilatus 200K CCD	AFC10: Fixed Chi 2 circle CCD
Absorption correction	Multi-scan *CrystalClear* (Rigaku, 2015 ▸)	Multi-scan *CrystalClear* (Rigaku, 2015 ▸)	Multi-scan *CrystalClear* (Rigaku, 2015 ▸)	Multi-scan *CrystalClear* (Rigaku, 2015 ▸)	Multi-scan
*T*_min_, *T*_max_	0.535, 1.000	0.783, 1.000	0.505, 1.000	0.744, 1.000	0.851, 1.000
No. of measured, independent and observed [*I* > 2σ(*I*)] reflections	22221, 1729, 1644	50824, 3308, 3177	13254, 1630, 1599	11869, 4842, 4831	28291, 3383, 3172
*R*_int_	0.045	0.033	0.067	0.012	0.039
(sin θ/λ)_max_ (Å^−1^)	0.602	0.602	0.602	0.628	0.603

Refinement					
*R*[*F* ^2^ > 2σ(*F* ^2^)], *wR*(*F* ^2^), *S*	0.017, 0.048, 1.04	0.017, 0.049, 1.05	0.021, 0.051, 1.01	0.031, 0.085, 1.10	0.047, 0.111, 1.08
No. of reflections	1729	3308	1630	4842	3383
No. of parameters	133	249	125	343	203
H-atom treatment	H atoms treated by a mixture of independent and constrained refinement	H atoms treated by a mixture of independent and constrained refinement	H atoms treated by a mixture of independent and constrained refinement	H atoms treated by a mixture of independent and constrained refinement	H atoms treated by a mixture of independent and constrained refinement
Δρ_max_, Δρ_min_ (e Å^−3^)	0.27, −0.31	0.30, −0.40	0.59, −0.42	1.25, −0.67	1.10, −1.02
Absolute structure	–	–	Parsons *et al.*, 2013 ▸	–	–
Absolute structure parameter	–	–	0.011 (6)	–	–

Computer programs: *CrystalClear* (Rigaku, 2015 ▸), *SHELXS97* (Sheldrick, 2008 ▸), *SHELXL2018/3* (Sheldrick, 2015 ▸), *ORTEP-3 for Windows* (Farrugia, 2012 ▸) and *publCIF* (Westrip, 2010 ▸).

## supplementary crystallographic information

### Poly[[(2-amino-3-methylpyridine)-µ3-phosphonato-zinc] hemihydrate] (I) Crystal data

**Table:**

[Zn(HPO_3_)(C_6_H_8_N_2_)]·0.5H_2_O	*F*(000) = 1064
*M**_r_* = 525.00	*D*_x_ = 1.841 Mg m^−^^3^
Monoclinic, *C*2/*c*	Mo *K*α radiation, λ = 0.71073 Å
*a* = 23.282 (6) Å	Cell parameters from 2870 reflections
*b* = 5.1926 (1) Å	θ = 2.6–27.4°
*c* = 17.738 (5) Å	µ = 2.75 mm^−^^1^
β = 117.974 (4)°	*T* = 93 K
*V* = 1893.9 (7) Å^3^	Plate, colourless
*Z* = 4	0.20 × 0.02 × 0.02 mm

### Poly[[(2-amino-3-methylpyridine)-µ3-phosphonato-zinc] hemihydrate] (I) Data collection

**Table:**

Rigaku Pilatus 200K CCD diffractometer	1644 reflections with *I* > 2σ(*I*)
ω scans	*R*_int_ = 0.045
Absorption correction: multi-scan CrystalClear (Rigaku, 2015)	θ_max_ = 25.3°, θ_min_ = 2.0°
*T*_min_ = 0.535, *T*_max_ = 1.000	*h* = −28→28
22221 measured reflections	*k* = −6→6
1729 independent reflections	*l* = −21→21

### Poly[[(2-amino-3-methylpyridine)-µ3-phosphonato-zinc] hemihydrate] (I) Refinement

**Table:**

Refinement on *F*^2^	Primary atom site location: structure-invariant direct methods
Least-squares matrix: full	Hydrogen site location: mixed
*R*[*F*^2^ > 2σ(*F*^2^)] = 0.017	H atoms treated by a mixture of independent and constrained refinement
*wR*(*F*^2^) = 0.048	*w* = 1/[σ^2^(*F*_o_^2^) + (0.023*P*)^2^ + 2.3217*P*] where *P* = (*F*_o_^2^ + 2*F*_c_^2^)/3
*S* = 1.04	(Δ/σ)_max_ = 0.001
1729 reflections	Δρ_max_ = 0.27 e Å^−^^3^
133 parameters	Δρ_min_ = −0.31 e Å^−^^3^
0 restraints	

### Poly[[(2-amino-3-methylpyridine)-µ3-phosphonato-zinc] hemihydrate] (I) Special details

**Table:**

Geometry. All esds (except the esd in the dihedral angle between two l.s. planes) are estimated using the full covariance matrix. The cell esds are taken into account individually in the estimation of esds in distances, angles and torsion angles; correlations between esds in cell parameters are only used when they are defined by crystal symmetry. An approximate (isotropic) treatment of cell esds is used for estimating esds involving l.s. planes.

### Poly[[(2-amino-3-methylpyridine)-µ3-phosphonato-zinc] hemihydrate] (I) Fractional atomic coordinates and isotropic or equivalent isotropic displacement parameters (Å^2^)

**Table:**

	*x*	*y*	*z*	*U*_iso_*/*U*_eq_	
Zn1	0.57140 (2)	0.80209 (4)	0.58868 (2)	0.01314 (8)	
P1	0.57761 (2)	0.29299 (8)	0.49128 (3)	0.01281 (11)	
H1	0.623752	0.252550	0.469333	0.015*	
O1	0.57635 (6)	0.5813 (2)	0.50423 (8)	0.0230 (3)	
O2	0.51441 (6)	0.1968 (2)	0.41659 (7)	0.0201 (3)	
O3	0.59745 (6)	0.1434 (2)	0.57337 (7)	0.0202 (3)	
C1	0.62119 (8)	0.5143 (3)	0.75510 (10)	0.0155 (3)	
C2	0.66659 (8)	0.4595 (3)	0.84181 (10)	0.0178 (4)	
C3	0.72325 (8)	0.6005 (4)	0.87796 (10)	0.0210 (4)	
H3	0.754671	0.567006	0.935325	0.025*	
C4	0.73559 (8)	0.7932 (4)	0.83177 (11)	0.0198 (4)	
H4	0.774780	0.890002	0.856994	0.024*	
C5	0.68950 (8)	0.8368 (3)	0.74957 (11)	0.0167 (3)	
H5	0.697186	0.967793	0.717992	0.020*	
C6	0.65085 (10)	0.2538 (4)	0.88920 (12)	0.0232 (4)	
H6A	0.609698	0.295572	0.888912	0.035*	
H6B	0.685707	0.245000	0.948255	0.035*	
H6C	0.646912	0.087146	0.861266	0.035*	
N1	0.63290 (7)	0.7007 (3)	0.71073 (9)	0.0146 (3)	
N2	0.56459 (7)	0.3842 (3)	0.71611 (10)	0.0198 (3)	
H1N	0.5571 (10)	0.268 (4)	0.7377 (14)	0.024*	
H2N	0.5404 (10)	0.394 (4)	0.6633 (13)	0.024*	
O4	0.500000	0.0486 (4)	0.250000	0.0216 (4)	
H1O	0.5071 (11)	0.136 (4)	0.2897 (12)	0.026*	

### Poly[[(2-amino-3-methylpyridine)-µ3-phosphonato-zinc] hemihydrate] (I) Atomic displacement parameters (Å^2^)

**Table:**

	*U*^11^	*U*^22^	*U*^33^	*U*^12^	*U*^13^	*U*^23^
Zn1	0.01503 (12)	0.01152 (13)	0.01196 (12)	0.00098 (7)	0.00558 (9)	0.00153 (7)
P1	0.0145 (2)	0.0111 (2)	0.0136 (2)	0.00076 (16)	0.00731 (18)	0.00110 (15)
O1	0.0414 (8)	0.0122 (7)	0.0254 (6)	0.0024 (6)	0.0239 (6)	0.0024 (5)
O2	0.0155 (6)	0.0297 (8)	0.0135 (6)	0.0022 (5)	0.0056 (5)	−0.0024 (5)
O3	0.0264 (7)	0.0123 (6)	0.0157 (6)	−0.0028 (5)	0.0047 (5)	0.0016 (5)
C1	0.0194 (8)	0.0134 (8)	0.0171 (8)	0.0051 (7)	0.0112 (7)	0.0015 (7)
C2	0.0227 (8)	0.0166 (9)	0.0168 (8)	0.0083 (7)	0.0116 (7)	0.0041 (7)
C3	0.0200 (9)	0.0248 (10)	0.0147 (8)	0.0086 (8)	0.0053 (7)	0.0037 (7)
C4	0.0149 (8)	0.0211 (10)	0.0216 (9)	0.0014 (7)	0.0072 (7)	−0.0008 (7)
C5	0.0162 (8)	0.0160 (9)	0.0196 (8)	0.0016 (7)	0.0100 (7)	0.0018 (7)
C6	0.0305 (10)	0.0227 (10)	0.0192 (9)	0.0071 (8)	0.0141 (8)	0.0073 (7)
N1	0.0149 (7)	0.0145 (8)	0.0142 (7)	0.0019 (5)	0.0067 (6)	0.0023 (5)
N2	0.0213 (8)	0.0189 (8)	0.0189 (7)	−0.0026 (7)	0.0093 (6)	0.0067 (7)
O4	0.0314 (10)	0.0168 (10)	0.0210 (9)	0.000	0.0161 (8)	0.000

### Poly[[(2-amino-3-methylpyridine)-µ3-phosphonato-zinc] hemihydrate] (I) Geometric parameters (Å, º)

**Table:**

Zn1—O1	1.9324 (12)	C3—C4	1.405 (3)
Zn1—O3^i^	1.9333 (13)	C3—H3	0.9500
Zn1—O2^ii^	1.9557 (13)	C4—C5	1.364 (2)
Zn1—N1	2.0262 (14)	C4—H4	0.9500
P1—O1	1.5167 (13)	C5—N1	1.363 (2)
P1—O3	1.5183 (12)	C5—H5	0.9500
P1—O2	1.5301 (13)	C6—H6A	0.9800
P1—H1	1.3200	C6—H6B	0.9800
C1—N2	1.348 (2)	C6—H6C	0.9800
C1—N1	1.353 (2)	N2—H1N	0.78 (2)
C1—C2	1.428 (2)	N2—H2N	0.84 (2)
C2—C3	1.376 (3)	O4—H1O	0.79 (2)
C2—C6	1.507 (2)	O4—H1O^iii^	0.79 (2)
			
O1—Zn1—O3^i^	107.36 (5)	C2—C3—H3	119.3
O1—Zn1—O2^ii^	113.32 (5)	C4—C3—H3	119.3
O3^i^—Zn1—O2^ii^	111.79 (5)	C5—C4—C3	117.90 (16)
O1—Zn1—N1	113.89 (6)	C5—C4—H4	121.1
O3^i^—Zn1—N1	106.10 (5)	C3—C4—H4	121.1
O2^ii^—Zn1—N1	104.23 (5)	N1—C5—C4	122.92 (16)
O1—P1—O3	112.24 (7)	N1—C5—H5	118.5
O1—P1—O2	111.98 (7)	C4—C5—H5	118.5
O3—P1—O2	113.57 (7)	C2—C6—H6A	109.5
O1—P1—H1	106.1	C2—C6—H6B	109.5
O3—P1—H1	106.1	H6A—C6—H6B	109.5
O2—P1—H1	106.1	C2—C6—H6C	109.5
P1—O1—Zn1	135.67 (7)	H6A—C6—H6C	109.5
P1—O2—Zn1^ii^	125.89 (7)	H6B—C6—H6C	109.5
P1—O3—Zn1^iv^	129.00 (7)	C1—N1—C5	119.16 (14)
N2—C1—N1	118.38 (15)	C1—N1—Zn1	124.96 (11)
N2—C1—C2	120.29 (15)	C5—N1—Zn1	115.87 (11)
N1—C1—C2	121.32 (15)	C1—N2—H1N	121.9 (16)
C3—C2—C1	117.33 (15)	C1—N2—H2N	121.7 (14)
C3—C2—C6	123.23 (15)	H1N—N2—H2N	114 (2)
C1—C2—C6	119.44 (16)	H1O—O4—H1O^iii^	109 (3)
C2—C3—C4	121.36 (15)		
			
O3—P1—O1—Zn1	−19.79 (15)	C1—C2—C3—C4	0.8 (3)
O2—P1—O1—Zn1	109.32 (12)	C6—C2—C3—C4	−179.11 (16)
O1—P1—O2—Zn1^ii^	−65.88 (11)	C2—C3—C4—C5	0.0 (3)
O3—P1—O2—Zn1^ii^	62.53 (11)	C3—C4—C5—N1	−0.6 (3)
O1—P1—O3—Zn1^iv^	148.87 (9)	N2—C1—N1—C5	179.08 (15)
O2—P1—O3—Zn1^iv^	20.58 (12)	C2—C1—N1—C5	0.6 (2)
N2—C1—C2—C3	−179.58 (16)	N2—C1—N1—Zn1	0.1 (2)
N1—C1—C2—C3	−1.1 (2)	C2—C1—N1—Zn1	−178.42 (11)
N2—C1—C2—C6	0.3 (2)	C4—C5—N1—C1	0.3 (2)
N1—C1—C2—C6	178.77 (15)	C4—C5—N1—Zn1	179.41 (13)

Symmetry codes: (i) *x*, *y*+1, *z*; (ii) −*x*+1, −*y*+1, −*z*+1; (iii) −*x*+1, *y*, −*z*+1/2; (iv) *x*, *y*−1, *z*.

### Poly[[(2-amino-3-methylpyridine)-µ3-phosphonato-zinc] hemihydrate] (I) Hydrogen-bond geometry (Å, º)

**Table:**

*D*—H···*A*	*D*—H	H···*A*	*D*···*A*	*D*—H···*A*
N2—H1*N*···O4^v^	0.78 (2)	2.19 (2)	2.919 (2)	157 (2)
N2—H2*N*···O2^ii^	0.84 (2)	2.54 (2)	3.098 (2)	124.8 (18)
O4—H1*O*···O2	0.79 (2)	2.199 (19)	2.9165 (15)	152 (2)

Symmetry codes: (ii) −*x*+1, −*y*+1, −*z*+1; (v) −*x*+1, −*y*, −*z*+1.

### Poly[(2-amino-4-methylpyridine)-µ3-phosphonato-zinc] (II) Crystal data

**Table:**

[Zn(HPO_3_)(C_6_H_8_N_2_)]	*F*(000) = 2048
*M**_r_* = 253.49	*D*_x_ = 1.850 Mg m^−^^3^
Monoclinic, *C*2/*c*	Mo *K*α radiation, λ = 0.71073 Å
*a* = 22.873 (6) Å	Cell parameters from 5932 reflections
*b* = 12.307 (3) Å	θ = 2.0–27.4°
*c* = 16.633 (4) Å	µ = 2.85 mm^−^^1^
β = 128.954 (5)°	*T* = 93 K
*V* = 3641.1 (16) Å^3^	Rod, colourless
*Z* = 16	0.10 × 0.03 × 0.03 mm

### Poly[(2-amino-4-methylpyridine)-µ3-phosphonato-zinc] (II) Data collection

**Table:**

Rigaku Pilatus 200K CCD diffractometer	3177 reflections with *I* > 2σ(*I*)
ω scans	*R*_int_ = 0.033
Absorption correction: multi-scan CrystalClear (Rigaku, 2015)	θ_max_ = 25.3°, θ_min_ = 2.0°
*T*_min_ = 0.783, *T*_max_ = 1.000	*h* = −27→27
50824 measured reflections	*k* = −14→14
3308 independent reflections	*l* = −19→19

### Poly[(2-amino-4-methylpyridine)-µ3-phosphonato-zinc] (II) Refinement

**Table:**

Refinement on *F*^2^	Primary atom site location: structure-invariant direct methods
Least-squares matrix: full	Hydrogen site location: mixed
*R*[*F*^2^ > 2σ(*F*^2^)] = 0.017	H atoms treated by a mixture of independent and constrained refinement
*wR*(*F*^2^) = 0.049	*w* = 1/[σ^2^(*F*_o_^2^) + (0.0301*P*)^2^ + 3.9697*P*] where *P* = (*F*_o_^2^ + 2*F*_c_^2^)/3
*S* = 1.05	(Δ/σ)_max_ = 0.002
3308 reflections	Δρ_max_ = 0.30 e Å^−^^3^
249 parameters	Δρ_min_ = −0.40 e Å^−^^3^
0 restraints	

### Poly[(2-amino-4-methylpyridine)-µ3-phosphonato-zinc] (II) Special details

**Table:**

Geometry. All esds (except the esd in the dihedral angle between two l.s. planes) are estimated using the full covariance matrix. The cell esds are taken into account individually in the estimation of esds in distances, angles and torsion angles; correlations between esds in cell parameters are only used when they are defined by crystal symmetry. An approximate (isotropic) treatment of cell esds is used for estimating esds involving l.s. planes.

### Poly[(2-amino-4-methylpyridine)-µ3-phosphonato-zinc] (II) Fractional atomic coordinates and isotropic or equivalent isotropic displacement parameters (Å^2^)

**Table:**

	*x*	*y*	*z*	*U*_iso_*/*U*_eq_	
Zn1	0.13481 (2)	0.28075 (2)	0.46631 (2)	0.01035 (6)	
Zn2	0.38505 (2)	0.50195 (2)	0.68663 (2)	0.01038 (7)	
P1	0.30317 (2)	0.28259 (3)	0.65170 (3)	0.01005 (9)	
H1	0.342771	0.281106	0.753047	0.012*	
P2	0.46680 (2)	0.44040 (3)	0.59502 (3)	0.01048 (9)	
H2	0.443210	0.462953	0.501255	0.013*	
O1	0.22394 (6)	0.24425 (9)	0.60565 (8)	0.0126 (2)	
O2	0.30176 (6)	0.40043 (9)	0.62249 (8)	0.0142 (2)	
O3	0.34276 (6)	0.20612 (9)	0.62797 (8)	0.0133 (2)	
O4	0.42559 (6)	0.51766 (9)	0.61527 (9)	0.0151 (2)	
O5	0.55137 (6)	0.45868 (9)	0.66790 (8)	0.0141 (2)	
O6	0.44751 (6)	0.32169 (9)	0.59323 (9)	0.0192 (3)	
C1	0.02104 (9)	0.45553 (13)	0.39669 (12)	0.0139 (3)	
C2	−0.00238 (9)	0.56244 (13)	0.39473 (12)	0.0159 (3)	
H2A	−0.051858	0.585125	0.338516	0.019*	
C3	0.04591 (9)	0.63392 (13)	0.47361 (12)	0.0145 (3)	
C4	0.11895 (9)	0.59656 (13)	0.55592 (12)	0.0144 (3)	
H4	0.153296	0.642415	0.613212	0.017*	
C5	0.13951 (9)	0.49351 (12)	0.55196 (12)	0.0127 (3)	
H5	0.189116	0.470022	0.606940	0.015*	
C6	0.02238 (10)	0.74823 (14)	0.47273 (14)	0.0213 (4)	
H6A	0.063327	0.785428	0.536348	0.032*	
H6B	−0.022360	0.746660	0.468422	0.032*	
H6C	0.010814	0.787141	0.412832	0.032*	
N1	0.09243 (7)	0.42276 (10)	0.47310 (10)	0.0120 (3)	
N2	−0.02674 (9)	0.38312 (13)	0.32253 (12)	0.0209 (3)	
H1N	−0.0668 (13)	0.4006 (18)	0.2726 (17)	0.025*	
H2N	−0.0154 (12)	0.318 (2)	0.3279 (16)	0.025*	
C7	0.32300 (8)	0.68978 (13)	0.72502 (11)	0.0113 (3)	
C8	0.29315 (9)	0.79476 (12)	0.70867 (12)	0.0127 (3)	
H8	0.272479	0.816001	0.741065	0.015*	
C9	0.29372 (8)	0.86715 (13)	0.64575 (12)	0.0133 (3)	
C10	0.32631 (9)	0.83349 (13)	0.60142 (13)	0.0168 (3)	
H10	0.329589	0.881946	0.559930	0.020*	
C11	0.35344 (10)	0.72937 (13)	0.61886 (13)	0.0160 (3)	
H11	0.374707	0.706960	0.587550	0.019*	
C12	0.26058 (10)	0.97887 (14)	0.62570 (14)	0.0187 (3)	
H12A	0.274259	1.022946	0.590688	0.028*	
H12B	0.280148	1.013133	0.691512	0.028*	
H12C	0.205714	0.973379	0.581816	0.028*	
N3	0.35144 (7)	0.65672 (10)	0.67863 (10)	0.0118 (3)	
N4	0.32235 (8)	0.61703 (11)	0.78641 (11)	0.0151 (3)	
H3N	0.3081 (11)	0.6407 (16)	0.8213 (15)	0.018*	
H4N	0.3543 (12)	0.5680 (17)	0.8129 (15)	0.018*	

### Poly[(2-amino-4-methylpyridine)-µ3-phosphonato-zinc] (II) Atomic displacement parameters (Å^2^)

**Table:**

	*U*^11^	*U*^22^	*U*^33^	*U*^12^	*U*^13^	*U*^23^
Zn1	0.01047 (10)	0.00892 (10)	0.01329 (11)	−0.00061 (6)	0.00826 (9)	−0.00141 (6)
Zn2	0.01062 (11)	0.00830 (11)	0.01323 (11)	0.00089 (6)	0.00799 (9)	0.00035 (6)
P1	0.01020 (19)	0.0102 (2)	0.01011 (19)	0.00065 (14)	0.00655 (16)	0.00054 (14)
P2	0.01040 (19)	0.01063 (19)	0.01165 (19)	−0.00079 (14)	0.00753 (16)	−0.00177 (14)
O1	0.0124 (5)	0.0130 (5)	0.0136 (5)	0.0002 (4)	0.0087 (5)	0.0015 (4)
O2	0.0123 (5)	0.0115 (5)	0.0169 (5)	−0.0004 (4)	0.0082 (5)	0.0003 (4)
O3	0.0137 (5)	0.0138 (5)	0.0145 (5)	0.0029 (4)	0.0099 (5)	0.0022 (4)
O4	0.0182 (6)	0.0132 (5)	0.0200 (6)	0.0030 (5)	0.0150 (5)	0.0016 (4)
O5	0.0129 (5)	0.0150 (6)	0.0149 (5)	−0.0029 (4)	0.0089 (5)	−0.0027 (4)
O6	0.0164 (6)	0.0125 (6)	0.0312 (7)	−0.0044 (5)	0.0163 (5)	−0.0061 (5)
C1	0.0135 (7)	0.0160 (8)	0.0144 (7)	0.0000 (6)	0.0098 (7)	−0.0005 (6)
C2	0.0134 (8)	0.0175 (8)	0.0161 (8)	0.0046 (6)	0.0090 (7)	0.0034 (6)
C3	0.0186 (8)	0.0139 (8)	0.0184 (8)	0.0023 (6)	0.0152 (7)	0.0018 (6)
C4	0.0161 (8)	0.0139 (8)	0.0151 (8)	−0.0029 (6)	0.0107 (7)	−0.0036 (6)
C5	0.0099 (7)	0.0149 (8)	0.0126 (8)	−0.0004 (6)	0.0067 (7)	−0.0004 (6)
C6	0.0254 (9)	0.0146 (8)	0.0282 (10)	0.0050 (7)	0.0189 (8)	0.0013 (7)
N1	0.0117 (6)	0.0112 (6)	0.0142 (6)	−0.0001 (5)	0.0087 (6)	−0.0011 (5)
N2	0.0125 (7)	0.0188 (8)	0.0192 (8)	0.0004 (6)	0.0042 (6)	−0.0051 (6)
C7	0.0090 (7)	0.0122 (7)	0.0106 (7)	−0.0013 (6)	0.0052 (6)	−0.0011 (6)
C8	0.0128 (7)	0.0120 (7)	0.0146 (8)	0.0004 (6)	0.0093 (7)	−0.0015 (6)
C9	0.0125 (7)	0.0106 (7)	0.0145 (8)	−0.0010 (6)	0.0074 (6)	−0.0012 (6)
C10	0.0232 (8)	0.0124 (8)	0.0208 (8)	0.0018 (7)	0.0167 (7)	0.0037 (6)
C11	0.0213 (8)	0.0144 (8)	0.0200 (8)	0.0016 (6)	0.0167 (7)	0.0016 (6)
C12	0.0234 (9)	0.0121 (8)	0.0235 (9)	0.0043 (7)	0.0161 (8)	0.0023 (7)
N3	0.0130 (6)	0.0098 (6)	0.0139 (6)	0.0008 (5)	0.0092 (5)	0.0004 (5)
N4	0.0216 (7)	0.0120 (7)	0.0185 (7)	0.0043 (6)	0.0159 (6)	0.0037 (6)

### Poly[(2-amino-4-methylpyridine)-µ3-phosphonato-zinc] (II) Geometric parameters (Å, º)

**Table:**

Zn1—O3^i^	1.9391 (12)	C4—H4	0.9500
Zn1—O6^i^	1.9419 (12)	C5—N1	1.365 (2)
Zn1—O1	1.9463 (11)	C5—H5	0.9500
Zn1—N1	2.0358 (13)	C6—H6A	0.9800
Zn2—O4	1.9210 (12)	C6—H6B	0.9800
Zn2—O2	1.9423 (11)	C6—H6C	0.9800
Zn2—O5^ii^	1.9567 (12)	N2—H1N	0.79 (2)
Zn2—N3	2.0269 (14)	N2—H2N	0.82 (2)
P1—O3	1.5202 (11)	C7—N3	1.347 (2)
P1—O2	1.5234 (12)	C7—N4	1.365 (2)
P1—O1	1.5324 (12)	C7—C8	1.406 (2)
P1—H1	1.3200	C8—C9	1.381 (2)
P2—O4	1.5182 (12)	C8—H8	0.9500
P2—O6	1.5209 (12)	C9—C10	1.401 (2)
P2—O5	1.5211 (12)	C9—C12	1.503 (2)
P2—H2	1.3200	C10—C11	1.373 (2)
C1—N2	1.347 (2)	C10—H10	0.9500
C1—N1	1.355 (2)	C11—N3	1.359 (2)
C1—C2	1.413 (2)	C11—H11	0.9500
C2—C3	1.376 (2)	C12—H12A	0.9800
C2—H2A	0.9500	C12—H12B	0.9800
C3—C4	1.415 (2)	C12—H12C	0.9800
C3—C6	1.503 (2)	N4—H3N	0.88 (2)
C4—C5	1.368 (2)	N4—H4N	0.83 (2)
			
O3^i^—Zn1—O6^i^	107.84 (5)	N1—C5—C4	123.38 (15)
O3^i^—Zn1—O1	111.75 (5)	N1—C5—H5	118.3
O6^i^—Zn1—O1	114.29 (5)	C4—C5—H5	118.3
O3^i^—Zn1—N1	110.52 (5)	C3—C6—H6A	109.5
O6^i^—Zn1—N1	104.06 (5)	C3—C6—H6B	109.5
O1—Zn1—N1	108.11 (5)	H6A—C6—H6B	109.5
O4—Zn2—O2	114.40 (5)	C3—C6—H6C	109.5
O4—Zn2—O5^ii^	121.28 (5)	H6A—C6—H6C	109.5
O2—Zn2—O5^ii^	102.36 (5)	H6B—C6—H6C	109.5
O4—Zn2—N3	100.10 (5)	C1—N1—C5	117.98 (13)
O2—Zn2—N3	111.58 (5)	C1—N1—Zn1	122.59 (11)
O5^ii^—Zn2—N3	107.05 (5)	C5—N1—Zn1	119.05 (10)
O3—P1—O2	113.78 (6)	C1—N2—H1N	121.4 (16)
O3—P1—O1	112.94 (7)	C1—N2—H2N	121.4 (15)
O2—P1—O1	110.64 (6)	H1N—N2—H2N	117 (2)
O3—P1—H1	106.3	N3—C7—N4	117.81 (14)
O2—P1—H1	106.3	N3—C7—C8	121.16 (14)
O1—P1—H1	106.3	N4—C7—C8	121.02 (14)
O4—P2—O6	113.14 (7)	C9—C8—C7	120.32 (14)
O4—P2—O5	112.73 (7)	C9—C8—H8	119.8
O6—P2—O5	111.44 (6)	C7—C8—H8	119.8
O4—P2—H2	106.3	C8—C9—C10	118.03 (14)
O6—P2—H2	106.3	C8—C9—C12	120.98 (14)
O5—P2—H2	106.3	C10—C9—C12	120.99 (14)
P1—O1—Zn1	123.26 (6)	C11—C10—C9	118.96 (15)
P1—O2—Zn2	128.34 (7)	C11—C10—H10	120.5
P1—O3—Zn1^i^	131.45 (7)	C9—C10—H10	120.5
P2—O4—Zn2	133.02 (7)	N3—C11—C10	123.37 (15)
P2—O5—Zn2^ii^	127.58 (7)	N3—C11—H11	118.3
P2—O6—Zn1^i^	144.09 (8)	C10—C11—H11	118.3
N2—C1—N1	117.88 (15)	C9—C12—H12A	109.5
N2—C1—C2	121.14 (15)	C9—C12—H12B	109.5
N1—C1—C2	120.97 (14)	H12A—C12—H12B	109.5
C3—C2—C1	120.52 (15)	C9—C12—H12C	109.5
C3—C2—H2A	119.7	H12A—C12—H12C	109.5
C1—C2—H2A	119.7	H12B—C12—H12C	109.5
C2—C3—C4	117.82 (15)	C7—N3—C11	118.08 (14)
C2—C3—C6	121.41 (15)	C7—N3—Zn2	123.09 (10)
C4—C3—C6	120.77 (15)	C11—N3—Zn2	118.72 (11)
C5—C4—C3	119.16 (14)	C7—N4—H3N	117.5 (13)
C5—C4—H4	120.4	C7—N4—H4N	117.1 (14)
C3—C4—H4	120.4	H3N—N4—H4N	117.4 (19)
			
O3—P1—O1—Zn1	−87.69 (9)	N2—C1—N1—C5	−175.71 (14)
O2—P1—O1—Zn1	41.16 (9)	C2—C1—N1—C5	4.6 (2)
O3—P1—O2—Zn2	−69.80 (10)	N2—C1—N1—Zn1	11.4 (2)
O1—P1—O2—Zn2	161.80 (7)	C2—C1—N1—Zn1	−168.38 (11)
O2—P1—O3—Zn1^i^	−20.85 (11)	C4—C5—N1—C1	−2.1 (2)
O1—P1—O3—Zn1^i^	106.36 (9)	C4—C5—N1—Zn1	171.05 (12)
O6—P2—O4—Zn2	26.92 (12)	N3—C7—C8—C9	−1.2 (2)
O5—P2—O4—Zn2	−100.68 (10)	N4—C7—C8—C9	−179.53 (15)
O4—P2—O5—Zn2^ii^	35.10 (11)	C7—C8—C9—C10	−1.5 (2)
O6—P2—O5—Zn2^ii^	−93.38 (9)	C7—C8—C9—C12	178.71 (14)
O4—P2—O6—Zn1^i^	51.37 (15)	C8—C9—C10—C11	2.5 (2)
O5—P2—O6—Zn1^i^	179.64 (11)	C12—C9—C10—C11	−177.66 (15)
N2—C1—C2—C3	176.82 (15)	C9—C10—C11—N3	−1.0 (3)
N1—C1—C2—C3	−3.5 (2)	N4—C7—N3—C11	−178.87 (14)
C1—C2—C3—C4	−0.2 (2)	C8—C7—N3—C11	2.8 (2)
C1—C2—C3—C6	179.55 (15)	N4—C7—N3—Zn2	5.08 (19)
C2—C3—C4—C5	2.6 (2)	C8—C7—N3—Zn2	−173.28 (11)
C6—C3—C4—C5	−177.19 (15)	C10—C11—N3—C7	−1.7 (2)
C3—C4—C5—N1	−1.5 (2)	C10—C11—N3—Zn2	174.54 (13)

Symmetry codes: (i) −*x*+1/2, −*y*+1/2, −*z*+1; (ii) −*x*+1, *y*, −*z*+3/2.

### Poly[(2-amino-4-methylpyridine)-µ3-phosphonato-zinc] (II) Hydrogen-bond geometry (Å, º)

**Table:**

*D*—H···*A*	*D*—H	H···*A*	*D*···*A*	*D*—H···*A*
N2—H1*N*···O3^iii^	0.79 (2)	2.35 (2)	2.9056 (19)	128 (2)
N2—H2*N*···O6^i^	0.82 (2)	2.13 (2)	2.900 (2)	155 (2)
N4—H3*N*···O1^iv^	0.88 (2)	2.18 (2)	3.0310 (18)	163.7 (18)
N4—H4*N*···O5^ii^	0.83 (2)	2.39 (2)	3.1555 (19)	154.3 (18)
C5—H5···O2	0.95	2.57	3.315 (2)	136
C8—H8···O1^iv^	0.95	2.65	3.405 (2)	137
C11—H11···O4	0.95	2.51	3.105 (2)	120

Symmetry codes: (i) −*x*+1/2, −*y*+1/2, −*z*+1; (ii) −*x*+1, *y*, −*z*+3/2; (iii) *x*−1/2, −*y*+1/2, *z*−1/2; (iv) −*x*+1/2, *y*+1/2, −*z*+3/2.

### Poly[(2-amino-5-methylpyridine)-µ3-phosphonato-zinc] (III) Crystal data

**Table:**

[Zn(HPO_3_)(C_6_H_8_N_2_)]	*D*_x_ = 1.882 Mg m^−^^3^
*M**_r_* = 253.49	Mo *K*α radiation, λ = 0.71073 Å
Orthorhombic, *P*2_1_2_1_2_1_	Cell parameters from 3214 reflections
*a* = 5.1487 (9) Å	θ = 2.6–27.5°
*b* = 8.5316 (19) Å	µ = 2.90 mm^−^^1^
*c* = 20.371 (5) Å	*T* = 93 K
*V* = 894.8 (3) Å^3^	Prism, colourless
*Z* = 4	0.15 × 0.05 × 0.03 mm
*F*(000) = 512	

### Poly[(2-amino-5-methylpyridine)-µ3-phosphonato-zinc] (III) Data collection

**Table:**

Rigaku Pilatus 200K CCD diffractometer	1599 reflections with *I* > 2σ(*I*)
ω scans	*R*_int_ = 0.067
Absorption correction: multi-scan CrystalClear (Rigaku, 2015)	θ_max_ = 25.3°, θ_min_ = 2.6°
*T*_min_ = 0.505, *T*_max_ = 1.000	*h* = −6→6
13254 measured reflections	*k* = −10→10
1630 independent reflections	*l* = −23→24

### Poly[(2-amino-5-methylpyridine)-µ3-phosphonato-zinc] (III) Refinement

**Table:**

Refinement on *F*^2^	Hydrogen site location: mixed
Least-squares matrix: full	H atoms treated by a mixture of independent and constrained refinement
*R*[*F*^2^ > 2σ(*F*^2^)] = 0.021	*w* = 1/[σ^2^(*F*_o_^2^) + (0.0239*P*)^2^ + 0.0724*P*] where *P* = (*F*_o_^2^ + 2*F*_c_^2^)/3
*wR*(*F*^2^) = 0.051	(Δ/σ)_max_ = 0.001
*S* = 1.01	Δρ_max_ = 0.59 e Å^−^^3^
1630 reflections	Δρ_min_ = −0.41 e Å^−^^3^
125 parameters	Absolute structure: Parsons *et al.*, 2013
0 restraints	Absolute structure parameter: 0.011 (6)
Primary atom site location: dual	

### Poly[(2-amino-5-methylpyridine)-µ3-phosphonato-zinc] (III) Special details

**Table:**

Geometry. All esds (except the esd in the dihedral angle between two l.s. planes) are estimated using the full covariance matrix. The cell esds are taken into account individually in the estimation of esds in distances, angles and torsion angles; correlations between esds in cell parameters are only used when they are defined by crystal symmetry. An approximate (isotropic) treatment of cell esds is used for estimating esds involving l.s. planes.

### Poly[(2-amino-5-methylpyridine)-µ3-phosphonato-zinc] (III) Fractional atomic coordinates and isotropic or equivalent isotropic displacement parameters (Å^2^)

**Table:**

	*x*	*y*	*z*	*U*_iso_*/*U*_eq_	
Zn1	0.72989 (7)	0.60753 (3)	0.06019 (2)	0.01077 (13)	
P1	0.23596 (17)	0.80347 (7)	0.08606 (3)	0.01135 (19)	
H1	0.143314	0.860290	0.141598	0.014*	
O1	0.5244 (4)	0.7749 (2)	0.09764 (10)	0.0133 (5)	
O2	0.1793 (4)	0.9268 (2)	0.03353 (10)	0.0133 (4)	
O3	0.0960 (4)	0.6496 (2)	0.07460 (10)	0.0163 (5)	
C1	0.7698 (7)	0.2687 (3)	0.09670 (13)	0.0125 (6)	
C2	0.6882 (6)	0.1306 (3)	0.12868 (15)	0.0159 (7)	
H2	0.762594	0.032284	0.117423	0.019*	
C3	0.5011 (7)	0.1398 (3)	0.17599 (15)	0.0152 (6)	
H3	0.447008	0.046999	0.197875	0.018*	
C4	0.3865 (6)	0.2842 (3)	0.19301 (14)	0.0141 (6)	
C5	0.4702 (6)	0.4120 (3)	0.15785 (14)	0.0136 (6)	
H5	0.393376	0.510660	0.167355	0.016*	
C6	0.1796 (6)	0.2972 (3)	0.24478 (15)	0.0181 (7)	
H6A	0.040112	0.222380	0.235356	0.027*	
H6B	0.108871	0.403897	0.244860	0.027*	
H6C	0.254815	0.274000	0.287887	0.027*	
N1	0.6561 (5)	0.4067 (3)	0.11026 (11)	0.0116 (5)	
N2	0.9566 (5)	0.2636 (3)	0.04934 (13)	0.0168 (6)	
H2A	1.056 (7)	0.350 (4)	0.0429 (17)	0.020*	
H2B	1.045 (7)	0.189 (5)	0.0452 (17)	0.020*	

### Poly[(2-amino-5-methylpyridine)-µ3-phosphonato-zinc] (III) Atomic displacement parameters (Å^2^)

**Table:**

	*U*^11^	*U*^22^	*U*^33^	*U*^12^	*U*^13^	*U*^23^
Zn1	0.01207 (19)	0.0079 (2)	0.0123 (2)	−0.00022 (13)	−0.00027 (14)	0.00120 (10)
P1	0.0124 (4)	0.0098 (3)	0.0119 (4)	0.0014 (3)	−0.0003 (3)	0.0005 (3)
O1	0.0133 (11)	0.0114 (9)	0.0152 (11)	−0.0001 (9)	−0.0013 (9)	−0.0003 (8)
O2	0.0180 (11)	0.0108 (9)	0.0111 (10)	0.0021 (8)	−0.0004 (9)	0.0009 (8)
O3	0.0123 (11)	0.0137 (10)	0.0228 (11)	−0.0010 (8)	−0.0035 (10)	0.0024 (8)
C1	0.0160 (16)	0.0131 (12)	0.0085 (14)	−0.0009 (13)	−0.0046 (14)	−0.0004 (10)
C2	0.0206 (17)	0.0096 (13)	0.0175 (16)	0.0003 (12)	−0.0030 (14)	−0.0005 (11)
C3	0.0218 (15)	0.0101 (13)	0.0139 (15)	−0.0035 (12)	−0.0042 (13)	0.0033 (11)
C4	0.0159 (15)	0.0137 (14)	0.0128 (16)	−0.0024 (12)	−0.0036 (14)	0.0018 (11)
C5	0.0149 (15)	0.0108 (13)	0.0152 (16)	0.0008 (13)	−0.0022 (12)	−0.0020 (12)
C6	0.0184 (16)	0.0204 (15)	0.0156 (17)	−0.0026 (13)	0.0016 (13)	0.0014 (12)
N1	0.0151 (13)	0.0097 (10)	0.0101 (12)	0.0003 (10)	−0.0004 (10)	0.0006 (9)
N2	0.0190 (15)	0.0110 (13)	0.0205 (15)	0.0040 (11)	0.0026 (12)	−0.0010 (11)

### Poly[(2-amino-5-methylpyridine)-µ3-phosphonato-zinc] (III) Geometric parameters (Å, º)

**Table:**

Zn1—O1	1.9343 (19)	C2—H2	0.9500
Zn1—O3^i^	1.941 (2)	C3—C4	1.409 (4)
Zn1—O2^ii^	1.949 (2)	C3—H3	0.9500
Zn1—N1	2.030 (2)	C4—C5	1.374 (4)
P1—O3	1.515 (2)	C4—C6	1.503 (4)
P1—O1	1.523 (2)	C5—N1	1.363 (4)
P1—O2	1.529 (2)	C5—H5	0.9500
P1—H1	1.3200	C6—H6A	0.9800
C1—N1	1.344 (3)	C6—H6B	0.9800
C1—N2	1.363 (4)	C6—H6C	0.9800
C1—C2	1.411 (4)	N2—H2A	0.90 (4)
C2—C3	1.365 (5)	N2—H2B	0.79 (4)
			
O1—Zn1—O3^i^	109.56 (9)	C2—C3—C4	121.3 (3)
O1—Zn1—O2^ii^	115.09 (8)	C2—C3—H3	119.4
O3^i^—Zn1—O2^ii^	107.80 (9)	C4—C3—H3	119.4
O1—Zn1—N1	108.82 (9)	C5—C4—C3	115.8 (3)
O3^i^—Zn1—N1	105.19 (9)	C5—C4—C6	121.9 (3)
O2^ii^—Zn1—N1	109.90 (8)	C3—C4—C6	122.3 (3)
O3—P1—O1	110.43 (11)	N1—C5—C4	124.4 (3)
O3—P1—O2	113.41 (12)	N1—C5—H5	117.8
O1—P1—O2	113.86 (12)	C4—C5—H5	117.8
O3—P1—H1	106.2	C4—C6—H6A	109.5
O1—P1—H1	106.2	C4—C6—H6B	109.5
O2—P1—H1	106.2	H6A—C6—H6B	109.5
P1—O1—Zn1	126.17 (12)	C4—C6—H6C	109.5
P1—O2—Zn1^iii^	123.84 (11)	H6A—C6—H6C	109.5
P1—O3—Zn1^iv^	130.19 (13)	H6B—C6—H6C	109.5
N1—C1—N2	118.8 (2)	C1—N1—C5	118.8 (2)
N1—C1—C2	120.5 (3)	C1—N1—Zn1	123.69 (19)
N2—C1—C2	120.7 (2)	C5—N1—Zn1	117.45 (18)
C3—C2—C1	119.2 (3)	C1—N2—H2A	118 (2)
C3—C2—H2	120.4	C1—N2—H2B	121 (3)
C1—C2—H2	120.4	H2A—N2—H2B	108 (3)
			
O3—P1—O1—Zn1	31.32 (19)	C2—C3—C4—C6	179.8 (3)
O2—P1—O1—Zn1	−97.61 (15)	C3—C4—C5—N1	−1.6 (4)
O3—P1—O2—Zn1^iii^	−54.86 (18)	C6—C4—C5—N1	−179.7 (3)
O1—P1—O2—Zn1^iii^	72.54 (16)	N2—C1—N1—C5	−179.7 (3)
O1—P1—O3—Zn1^iv^	177.67 (14)	C2—C1—N1—C5	3.4 (4)
O2—P1—O3—Zn1^iv^	−53.2 (2)	N2—C1—N1—Zn1	4.0 (4)
N1—C1—C2—C3	−3.3 (5)	C2—C1—N1—Zn1	−172.9 (2)
N2—C1—C2—C3	179.9 (3)	C4—C5—N1—C1	−0.9 (4)
C1—C2—C3—C4	0.6 (5)	C4—C5—N1—Zn1	175.6 (2)
C2—C3—C4—C5	1.7 (5)		

Symmetry codes: (i) *x*+1, *y*, *z*; (ii) *x*+1/2, −*y*+3/2, −*z*; (iii) *x*−1/2, −*y*+3/2, −*z*; (iv) *x*−1, *y*, *z*.

### Poly[(2-amino-5-methylpyridine)-µ3-phosphonato-zinc] (III) Hydrogen-bond geometry (Å, º)

**Table:**

*D*—H···*A*	*D*—H	H···*A*	*D*···*A*	*D*—H···*A*
N2—H2*B*···O2^v^	0.79 (4)	2.35 (4)	3.111 (3)	162 (3)
C2—H2···O1^vi^	0.95	2.55	3.212 (3)	127

Symmetry codes: (v) *x*+1, *y*−1, *z*; (vi) *x*, *y*−1, *z*.

### Poly[bis(2-amino-4-methylpyridinium) [tetra-µ3-phosphonato-trizinc] monohydrate] (IV) Crystal data

**Table:**

(C_6_H_9_N_2_)_2_[Zn_3_(HPO_3_)_4_]·H_2_O	*Z* = 2
*M**_r_* = 752.34	*F*(000) = 756
Triclinic, *P*1	*D*_x_ = 2.019 Mg m^−^^3^
*a* = 9.03805 (13) Å	Cu *K*α radiation, λ = 1.54184 Å
*b* = 9.36837 (13) Å	Cell parameters from 10623 reflections
*c* = 15.0207 (2) Å	θ = 5.7–75.3°
α = 81.0313 (1)°	µ = 6.49 mm^−^^1^
β = 88.0646 (1)°	*T* = 93 K
γ = 80.0782 (1)°	Prism, colourless
*V* = 1237.46 (3) Å^3^	0.10 × 0.10 × 0.10 mm

### Poly[bis(2-amino-4-methylpyridinium) [tetra-µ3-phosphonato-trizinc] monohydrate] (IV) Data collection

**Table:**

Rigaku Pilatus 200K CCD diffractometer	4842 independent reflections
Radiation source: fine-focus sealed X-ray tube	4831 reflections with *I* > 2σ(*I*)
Graphite monochromator	*R*_int_ = 0.012
ω scans	θ_max_ = 75.4°, θ_min_ = 3.0°
Absorption correction: multi-scan CrystalClear (Rigaku, 2015)	*h* = −11→11
*T*_min_ = 0.744, *T*_max_ = 1.000	*k* = −11→11
11869 measured reflections	*l* = −18→18

### Poly[bis(2-amino-4-methylpyridinium) [tetra-µ3-phosphonato-trizinc] monohydrate] (IV) Refinement

**Table:**

Refinement on *F*^2^	Hydrogen site location: mixed
Least-squares matrix: full	H atoms treated by a mixture of independent and constrained refinement
*R*[*F*^2^ > 2σ(*F*^2^)] = 0.031	*w* = 1/[σ^2^(*F*_o_^2^) + (0.0455*P*)^2^ + 2.3807*P*] where *P* = (*F*_o_^2^ + 2*F*_c_^2^)/3
*wR*(*F*^2^) = 0.085	(Δ/σ)_max_ = 0.001
*S* = 1.10	Δρ_max_ = 1.25 e Å^−^^3^
4842 reflections	Δρ_min_ = −0.67 e Å^−^^3^
343 parameters	Extinction correction: SHELXL-2018/3 (Sheldrick 2015), Fc^*^=kFc[1+0.001xFc^2^λ^3^/sin(2θ)]^-1/4^
0 restraints	Extinction coefficient: 0.0053 (3)
Primary atom site location: structure-invariant direct methods	

### Poly[bis(2-amino-4-methylpyridinium) [tetra-µ3-phosphonato-trizinc] monohydrate] (IV) Special details

**Table:**

Geometry. All esds (except the esd in the dihedral angle between two l.s. planes) are estimated using the full covariance matrix. The cell esds are taken into account individually in the estimation of esds in distances, angles and torsion angles; correlations between esds in cell parameters are only used when they are defined by crystal symmetry. An approximate (isotropic) treatment of cell esds is used for estimating esds involving l.s. planes.

### Poly[bis(2-amino-4-methylpyridinium) [tetra-µ3-phosphonato-trizinc] monohydrate] (IV) Fractional atomic coordinates and isotropic or equivalent isotropic displacement parameters (Å^2^)

**Table:**

	*x*	*y*	*z*	*U*_iso_*/*U*_eq_	
Zn1	0.24743 (4)	0.40103 (4)	0.26283 (2)	0.01319 (11)	
Zn2	−0.39686 (4)	0.53650 (4)	0.37317 (2)	0.01450 (11)	
Zn3	0.36057 (4)	0.44962 (4)	−0.08606 (2)	0.01489 (11)	
P1	−0.11180 (7)	0.49421 (7)	0.23815 (4)	0.01223 (14)	
H1	−0.085208	0.618572	0.259906	0.015*	
O1	0.0349 (2)	0.3858 (2)	0.24886 (13)	0.0181 (4)	
O2	−0.2305 (2)	0.4425 (2)	0.30286 (13)	0.0194 (4)	
O3	−0.1634 (2)	0.5258 (2)	0.14074 (12)	0.0198 (4)	
P2	0.43840 (7)	0.28650 (7)	0.10450 (4)	0.01276 (14)	
H2	0.489858	0.157007	0.080888	0.015*	
O4	0.3711 (2)	0.2551 (2)	0.19875 (12)	0.0175 (4)	
O5	0.3142 (2)	0.3593 (2)	0.03697 (12)	0.0172 (4)	
O6	0.5709 (2)	0.3653 (2)	0.09860 (13)	0.0196 (4)	
P3	0.39182 (7)	0.30396 (7)	0.46061 (4)	0.01410 (15)	
H3	0.431225	0.160024	0.476842	0.017*	
O7	0.2721 (2)	0.3341 (2)	0.38856 (12)	0.0223 (4)	
O8	0.3247 (2)	0.3488 (2)	0.54810 (12)	0.0219 (4)	
O9	0.5344 (2)	0.3632 (2)	0.43166 (14)	0.0248 (4)	
P4	0.38437 (7)	0.69938 (7)	0.20856 (4)	0.01361 (15)	
H4	0.303842	0.832430	0.198557	0.016*	
O10	0.2702 (2)	0.5969 (2)	0.20914 (14)	0.0220 (4)	
O11	0.4872 (2)	0.6985 (2)	0.12705 (13)	0.0237 (4)	
O12	0.4649 (2)	0.6836 (2)	0.29693 (13)	0.0240 (4)	
C1	0.0589 (3)	0.7635 (3)	−0.01704 (17)	0.0150 (5)	
C2	0.1635 (3)	0.8612 (3)	−0.03501 (17)	0.0162 (5)	
H2A	0.251603	0.845508	0.000413	0.019*	
C3	0.1385 (3)	0.9781 (3)	−0.10321 (18)	0.0171 (5)	
C4	0.0059 (3)	0.9998 (3)	−0.15470 (18)	0.0205 (5)	
H4A	−0.014596	1.081128	−0.201535	0.025*	
C5	−0.0916 (3)	0.9041 (3)	−0.13684 (18)	0.0199 (5)	
H5	−0.179518	0.917499	−0.172217	0.024*	
C6	0.2478 (3)	1.0834 (3)	−0.1230 (2)	0.0228 (6)	
H6A	0.193054	1.184235	−0.129661	0.034*	
H6B	0.302513	1.067140	−0.178991	0.034*	
H6C	0.319166	1.067367	−0.073283	0.034*	
N1	−0.0650 (2)	0.7888 (2)	−0.06862 (15)	0.0170 (4)	
H1A	−0.130665	0.728796	−0.057878	0.020*	
N2	0.0770 (3)	0.6485 (3)	0.04803 (16)	0.0198 (5)	
H2N	0.008 (4)	0.598 (4)	0.063 (2)	0.024*	
H3N	0.152 (4)	0.637 (4)	0.083 (3)	0.024*	
C7	−0.1126 (4)	0.0499 (3)	0.3638 (2)	0.0293 (6)	
C8	−0.0951 (4)	−0.1046 (3)	0.3921 (2)	0.0298 (7)	
H8	−0.178225	−0.153971	0.391019	0.036*	
C9	0.0422 (4)	−0.1811 (3)	0.4206 (2)	0.0269 (6)	
C10	0.1682 (4)	−0.1098 (4)	0.4158 (2)	0.0312 (7)	
H10	0.264069	−0.162712	0.434784	0.037*	
C11	0.1501 (4)	0.0345 (4)	0.3839 (2)	0.0357 (7)	
H11	0.235379	0.082043	0.376766	0.043*	
C12	0.0592 (4)	−0.3416 (3)	0.4520 (2)	0.0320 (7)	
H12A	0.094069	−0.363281	0.514548	0.048*	
H12B	0.132633	−0.393645	0.413711	0.048*	
H12C	−0.037868	−0.373520	0.448605	0.048*	
N3	0.0130 (3)	0.1129 (3)	0.3619 (2)	0.0287 (6)	
H4N	0.011 (5)	0.199 (5)	0.338 (3)	0.034*	
N4	−0.2409 (3)	0.1292 (3)	0.3372 (2)	0.0290 (6)	
H5N	−0.321 (5)	0.081 (5)	0.333 (3)	0.035*	
H6N	−0.249 (5)	0.225 (5)	0.321 (3)	0.035*	
O13	0.4957 (3)	0.0103 (2)	−0.32132 (16)	0.0327 (5)	
H1O	0.511797	0.109711	−0.317211	0.039*	
H2O	0.552497	−0.056389	−0.275011	0.039*	

### Poly[bis(2-amino-4-methylpyridinium) [tetra-µ3-phosphonato-trizinc] monohydrate] (IV) Atomic displacement parameters (Å^2^)

**Table:**

	*U*^11^	*U*^22^	*U*^33^	*U*^12^	*U*^13^	*U*^23^
Zn1	0.01172 (17)	0.01579 (18)	0.01205 (17)	−0.00462 (13)	−0.00061 (12)	0.00056 (12)
Zn2	0.01256 (18)	0.01957 (19)	0.01204 (17)	−0.00494 (13)	0.00048 (12)	−0.00209 (13)
Zn3	0.01393 (18)	0.01878 (19)	0.01254 (17)	−0.00717 (13)	−0.00082 (12)	0.00086 (13)
P1	0.0117 (3)	0.0135 (3)	0.0114 (3)	−0.0041 (2)	−0.0004 (2)	0.0008 (2)
O1	0.0112 (8)	0.0185 (9)	0.0246 (9)	−0.0046 (7)	−0.0014 (7)	−0.0009 (7)
O2	0.0194 (9)	0.0190 (9)	0.0189 (9)	−0.0047 (7)	0.0076 (7)	−0.0003 (7)
O3	0.0169 (9)	0.0286 (10)	0.0140 (9)	−0.0101 (8)	−0.0033 (7)	0.0039 (7)
P2	0.0117 (3)	0.0135 (3)	0.0128 (3)	−0.0031 (2)	0.0008 (2)	−0.0003 (2)
O4	0.0191 (9)	0.0186 (9)	0.0130 (8)	−0.0021 (7)	0.0018 (7)	0.0016 (7)
O5	0.0139 (8)	0.0230 (9)	0.0137 (8)	−0.0048 (7)	−0.0006 (7)	0.0018 (7)
O6	0.0141 (9)	0.0189 (9)	0.0264 (10)	−0.0062 (7)	−0.0004 (7)	−0.0012 (7)
P3	0.0157 (3)	0.0147 (3)	0.0121 (3)	−0.0053 (2)	0.0006 (2)	0.0002 (2)
O7	0.0250 (10)	0.0290 (10)	0.0132 (9)	−0.0081 (8)	−0.0028 (7)	0.0005 (7)
O8	0.0223 (10)	0.0330 (11)	0.0144 (9)	−0.0137 (8)	0.0026 (7)	−0.0065 (8)
O9	0.0196 (10)	0.0237 (10)	0.0314 (11)	−0.0089 (8)	0.0077 (8)	−0.0005 (8)
P4	0.0127 (3)	0.0129 (3)	0.0149 (3)	−0.0027 (2)	−0.0015 (2)	−0.0002 (2)
O10	0.0180 (9)	0.0197 (10)	0.0285 (10)	−0.0089 (8)	−0.0042 (8)	0.0031 (8)
O11	0.0281 (10)	0.0206 (10)	0.0220 (10)	−0.0060 (8)	0.0082 (8)	−0.0013 (8)
O12	0.0259 (10)	0.0257 (10)	0.0202 (10)	0.0003 (8)	−0.0084 (8)	−0.0062 (8)
C1	0.0140 (11)	0.0154 (12)	0.0148 (11)	−0.0006 (9)	0.0019 (9)	−0.0026 (9)
C2	0.0130 (11)	0.0180 (12)	0.0177 (12)	−0.0025 (10)	0.0006 (9)	−0.0031 (10)
C3	0.0159 (12)	0.0154 (12)	0.0200 (12)	−0.0032 (10)	0.0049 (10)	−0.0034 (10)
C4	0.0234 (14)	0.0173 (13)	0.0189 (12)	−0.0024 (11)	−0.0004 (10)	0.0021 (10)
C5	0.0186 (13)	0.0200 (13)	0.0201 (13)	−0.0027 (10)	−0.0053 (10)	0.0002 (10)
C6	0.0184 (13)	0.0176 (13)	0.0320 (15)	−0.0065 (11)	0.0049 (11)	0.0002 (11)
N1	0.0153 (10)	0.0165 (11)	0.0196 (11)	−0.0053 (8)	−0.0007 (8)	−0.0007 (8)
N2	0.0174 (11)	0.0223 (12)	0.0190 (11)	−0.0069 (9)	−0.0011 (9)	0.0038 (9)
C7	0.0239 (15)	0.0257 (15)	0.0362 (17)	−0.0043 (12)	0.0044 (12)	0.0008 (12)
C8	0.0315 (16)	0.0264 (15)	0.0319 (16)	−0.0093 (13)	0.0069 (13)	−0.0021 (12)
C9	0.0274 (15)	0.0214 (14)	0.0311 (15)	−0.0061 (12)	0.0042 (12)	−0.0005 (11)
C10	0.0245 (15)	0.0275 (16)	0.0416 (18)	−0.0047 (12)	−0.0027 (13)	−0.0046 (13)
C11	0.0346 (18)	0.0373 (18)	0.0385 (18)	−0.0144 (15)	0.0015 (14)	−0.0074 (14)
C12	0.0360 (17)	0.0199 (15)	0.0385 (17)	−0.0029 (13)	−0.0012 (14)	−0.0008 (12)
N3	0.0274 (13)	0.0162 (12)	0.0421 (15)	−0.0099 (10)	0.0027 (11)	0.0031 (11)
N4	0.0247 (13)	0.0191 (13)	0.0415 (15)	−0.0042 (10)	0.0023 (11)	0.0010 (11)
O13	0.0342 (12)	0.0281 (11)	0.0355 (12)	−0.0088 (10)	−0.0085 (10)	0.0016 (9)

### Poly[bis(2-amino-4-methylpyridinium) [tetra-µ3-phosphonato-trizinc] monohydrate] (IV) Geometric parameters (Å, º)

**Table:**

Zn1—O7	1.9028 (19)	C2—H2A	0.9500
Zn1—O10	1.9283 (19)	C3—C4	1.416 (4)
Zn1—O4	1.9637 (19)	C3—C6	1.504 (4)
Zn1—O1	1.9710 (18)	C4—C5	1.354 (4)
Zn2—O9^i^	1.913 (2)	C4—H4A	0.9500
Zn2—O8^ii^	1.9154 (19)	C5—N1	1.363 (3)
Zn2—O12^i^	1.945 (2)	C5—H5	0.9500
Zn2—O2	1.9690 (19)	C6—H6A	0.9800
Zn3—O6^iii^	1.9202 (19)	C6—H6B	0.9800
Zn3—O11^iii^	1.936 (2)	C6—H6C	0.9800
Zn3—O3^iv^	1.9499 (18)	N1—H1A	0.8800
Zn3—O5	1.9720 (18)	N2—H2N	0.85 (4)
P1—O2	1.5142 (18)	N2—H3N	0.85 (4)
P1—O3	1.5196 (18)	C7—N4	1.304 (4)
P1—O1	1.5217 (19)	C7—N3	1.364 (4)
P1—H1	1.3200	C7—C8	1.426 (4)
P2—O6	1.5043 (19)	C8—C9	1.368 (5)
P2—O4	1.5279 (18)	C8—H8	0.9500
P2—O5	1.5333 (18)	C9—C10	1.411 (4)
P2—H2	1.3200	C9—C12	1.486 (4)
P3—O9	1.514 (2)	C10—C11	1.346 (5)
P3—O7	1.517 (2)	C10—H10	0.9500
P3—O8	1.5172 (19)	C11—N3	1.350 (4)
P3—H3	1.3200	C11—H11	0.9500
P4—O12	1.5103 (19)	C12—H12A	0.9800
P4—O11	1.512 (2)	C12—H12B	0.9800
P4—O10	1.5247 (19)	C12—H12C	0.9800
P4—H4	1.3200	N3—H4N	0.83 (4)
C1—N2	1.329 (3)	N4—H5N	0.93 (4)
C1—N1	1.349 (3)	N4—H6N	0.88 (4)
C1—C2	1.417 (4)	O13—H1O	0.9780
C2—C3	1.372 (4)	O13—H2O	0.9570
			
O7—Zn1—O10	122.18 (9)	N2—C1—C2	123.2 (2)
O7—Zn1—O4	107.57 (8)	N1—C1—C2	118.3 (2)
O10—Zn1—O4	110.71 (8)	C3—C2—C1	120.4 (2)
O7—Zn1—O1	100.42 (8)	C3—C2—H2A	119.8
O10—Zn1—O1	106.69 (8)	C1—C2—H2A	119.8
O4—Zn1—O1	108.12 (8)	C2—C3—C4	118.8 (2)
O9^i^—Zn2—O8^ii^	115.30 (9)	C2—C3—C6	121.2 (2)
O9^i^—Zn2—O12^i^	118.93 (9)	C4—C3—C6	120.0 (2)
O8^ii^—Zn2—O12^i^	102.04 (9)	C5—C4—C3	119.7 (2)
O9^i^—Zn2—O2	98.33 (8)	C5—C4—H4A	120.2
O8^ii^—Zn2—O2	110.78 (8)	C3—C4—H4A	120.2
O12^i^—Zn2—O2	111.74 (8)	C4—C5—N1	120.7 (2)
O6^iii^—Zn3—O11^iii^	110.85 (8)	C4—C5—H5	119.7
O6^iii^—Zn3—O3^iv^	110.21 (8)	N1—C5—H5	119.7
O11^iii^—Zn3—O3^iv^	115.57 (9)	C3—C6—H6A	109.5
O6^iii^—Zn3—O5	117.10 (8)	C3—C6—H6B	109.5
O11^iii^—Zn3—O5	103.07 (8)	H6A—C6—H6B	109.5
O3^iv^—Zn3—O5	99.70 (8)	C3—C6—H6C	109.5
O2—P1—O3	112.70 (11)	H6A—C6—H6C	109.5
O2—P1—O1	111.00 (11)	H6B—C6—H6C	109.5
O3—P1—O1	111.15 (11)	C1—N1—C5	122.1 (2)
O2—P1—H1	107.2	C1—N1—H1A	118.9
O3—P1—H1	107.2	C5—N1—H1A	118.9
O1—P1—H1	107.2	C1—N2—H2N	122 (2)
P1—O1—Zn1	135.03 (12)	C1—N2—H3N	118 (2)
P1—O2—Zn2	135.93 (12)	H2N—N2—H3N	119 (3)
P1—O3—Zn3^iv^	132.51 (12)	N4—C7—N3	120.2 (3)
O6—P2—O4	115.33 (11)	N4—C7—C8	122.4 (3)
O6—P2—O5	113.41 (11)	N3—C7—C8	117.3 (3)
O4—P2—O5	110.08 (10)	C9—C8—C7	119.6 (3)
O6—P2—H2	105.7	C9—C8—H8	120.2
O4—P2—H2	105.7	C7—C8—H8	120.2
O5—P2—H2	105.7	C8—C9—C10	120.2 (3)
P2—O4—Zn1	125.40 (11)	C8—C9—C12	119.5 (3)
P2—O5—Zn3	121.78 (11)	C10—C9—C12	120.2 (3)
P2—O6—Zn3^iii^	146.90 (13)	C11—C10—C9	118.8 (3)
O9—P3—O7	114.49 (12)	C11—C10—H10	120.6
O9—P3—O8	113.97 (12)	C9—C10—H10	120.6
O7—P3—O8	110.19 (11)	C10—C11—N3	121.3 (3)
O9—P3—H3	105.8	C10—C11—H11	119.4
O7—P3—H3	105.8	N3—C11—H11	119.4
O8—P3—H3	105.8	C9—C12—H12A	109.5
P3—O7—Zn1	140.37 (13)	C9—C12—H12B	109.5
P3—O8—Zn2^ii^	129.47 (12)	H12A—C12—H12B	109.5
P3—O9—Zn2^v^	141.65 (14)	C9—C12—H12C	109.5
O12—P4—O11	114.13 (12)	H12A—C12—H12C	109.5
O12—P4—O10	114.03 (12)	H12B—C12—H12C	109.5
O11—P4—O10	112.68 (12)	C11—N3—C7	122.5 (3)
O12—P4—H4	104.9	C11—N3—H4N	116 (3)
O11—P4—H4	104.9	C7—N3—H4N	121 (3)
O10—P4—H4	104.9	C7—N4—H5N	118 (3)
P4—O10—Zn1	138.13 (12)	C7—N4—H6N	121 (3)
P4—O11—Zn3^iii^	133.31 (12)	H5N—N4—H6N	122 (4)
P4—O12—Zn2^v^	138.52 (13)	H1O—O13—H2O	108.6
N2—C1—N1	118.5 (2)		
			
O2—P1—O1—Zn1	−131.13 (16)	O11—P4—O12—Zn2^v^	−67.0 (2)
O3—P1—O1—Zn1	102.59 (17)	O10—P4—O12—Zn2^v^	64.4 (2)
O3—P1—O2—Zn2	−89.87 (19)	N2—C1—C2—C3	180.0 (2)
O1—P1—O2—Zn2	144.71 (16)	N1—C1—C2—C3	0.1 (4)
O2—P1—O3—Zn3^iv^	14.2 (2)	C1—C2—C3—C4	0.4 (4)
O1—P1—O3—Zn3^iv^	139.51 (15)	C1—C2—C3—C6	179.7 (2)
O6—P2—O4—Zn1	74.23 (16)	C2—C3—C4—C5	−1.1 (4)
O5—P2—O4—Zn1	−55.63 (16)	C6—C3—C4—C5	179.6 (3)
O6—P2—O5—Zn3	37.41 (16)	C3—C4—C5—N1	1.3 (4)
O4—P2—O5—Zn3	168.29 (11)	N2—C1—N1—C5	−179.8 (2)
O4—P2—O6—Zn3^iii^	−85.7 (2)	C2—C1—N1—C5	0.1 (4)
O5—P2—O6—Zn3^iii^	42.5 (3)	C4—C5—N1—C1	−0.8 (4)
O9—P3—O7—Zn1	12.1 (3)	N4—C7—C8—C9	−179.0 (3)
O8—P3—O7—Zn1	142.09 (19)	N3—C7—C8—C9	3.7 (5)
O9—P3—O8—Zn2^ii^	−1.7 (2)	C7—C8—C9—C10	−4.7 (5)
O7—P3—O8—Zn2^ii^	−132.07 (16)	C7—C8—C9—C12	178.6 (3)
O7—P3—O9—Zn2^v^	47.6 (3)	C8—C9—C10—C11	0.9 (5)
O8—P3—O9—Zn2^v^	−80.5 (2)	C12—C9—C10—C11	177.5 (3)
O12—P4—O10—Zn1	−34.6 (2)	C9—C10—C11—N3	4.1 (5)
O11—P4—O10—Zn1	97.6 (2)	C10—C11—N3—C7	−5.2 (5)
O12—P4—O11—Zn3^iii^	66.5 (2)	N4—C7—N3—C11	−176.2 (3)
O10—P4—O11—Zn3^iii^	−65.6 (2)	C8—C7—N3—C11	1.2 (5)

Symmetry codes: (i) *x*−1, *y*, *z*; (ii) −*x*, −*y*+1, −*z*+1; (iii) −*x*+1, −*y*+1, −*z*; (iv) −*x*, −*y*+1, −*z*; (v) *x*+1, *y*, *z*.

### Poly[bis(2-amino-4-methylpyridinium) [tetra-µ3-phosphonato-trizinc] monohydrate] (IV) Hydrogen-bond geometry (Å, º)

**Table:**

*D*—H···*A*	*D*—H	H···*A*	*D*···*A*	*D*—H···*A*
N1—H1*A*···O5^iv^	0.88	1.97	2.833 (3)	166
N2—H2*N*···O3	0.85 (4)	2.05 (4)	2.853 (3)	157 (3)
N2—H3*N*···O10	0.85 (4)	2.16 (4)	2.961 (3)	156 (3)
N3—H4*N*···O1	0.83 (4)	2.07 (4)	2.873 (3)	163 (4)
N4—H5*N*···O13^vi^	0.93 (4)	1.95 (4)	2.871 (4)	177 (4)
N4—H6*N*···O2	0.88 (4)	2.05 (4)	2.918 (3)	167 (4)
O13—H1*O*···O12^iii^	0.98	2.05	3.028 (3)	175
O13—H2*O*···O4^vii^	0.96	2.05	2.952 (3)	157
C5—H5···O4^iv^	0.95	2.64	3.370 (3)	134
C8—H8···O8^viii^	0.95	2.48	3.350 (4)	152
C11—H11···O7	0.95	2.47	3.199 (4)	133
C11—H11···O13^vii^	0.95	2.59	3.281 (4)	130

Symmetry codes: (iii) −*x*+1, −*y*+1, −*z*; (iv) −*x*, −*y*+1, −*z*; (vi) −*x*, −*y*, −*z*; (vii) −*x*+1, −*y*, −*z*; (viii) −*x*, −*y*, −*z*+1.

### (V) Crystal data

**Table:**

(C_6_H_9_N_2_)_2_[ZnCl_4_]·H_2_O	*Z* = 2
*M**_r_* = 443.49	*F*(000) = 452
Triclinic, *P*1	*D*_x_ = 1.596 Mg m^−^^3^
*a* = 6.9541 (8) Å	Mo *K*α radiation, λ = 0.71073 Å
*b* = 8.7092 (9) Å	Cell parameters from 3331 reflections
*c* = 16.293 (3) Å	θ = 2.5–27.4°
α = 83.239 (11)°	µ = 1.92 mm^−^^1^
β = 80.167 (10)°	*T* = 173 K
γ = 72.049 (9)°	Prism, colourless
*V* = 922.7 (2) Å^3^	0.15 × 0.05 × 0.05 mm

### (V) Data collection

**Table:**

AFC10: Fixed Chi 2 circle CCD diffractometer	3383 independent reflections
Radiation source: Rotating Anode	3172 reflections with *I* > 2σ(*I*)
Confocal monochromator	*R*_int_ = 0.039
ω scans	θ_max_ = 25.4°, θ_min_ = 2.5°
Absorption correction: multi-scan	*h* = −8→8
*T*_min_ = 0.851, *T*_max_ = 1.000	*k* = −10→10
28291 measured reflections	*l* = −19→19

### (V) Refinement

**Table:**

Refinement on *F*^2^	Primary atom site location: structure-invariant direct methods
Least-squares matrix: full	Hydrogen site location: mixed
*R*[*F*^2^ > 2σ(*F*^2^)] = 0.047	H atoms treated by a mixture of independent and constrained refinement
*wR*(*F*^2^) = 0.111	*w* = 1/[σ^2^(*F*_o_^2^) + (0.0281*P*)^2^ + 3.4309*P*] where *P* = (*F*_o_^2^ + 2*F*_c_^2^)/3
*S* = 1.08	(Δ/σ)_max_ = 0.001
3383 reflections	Δρ_max_ = 1.10 e Å^−^^3^
203 parameters	Δρ_min_ = −1.01 e Å^−^^3^
0 restraints	

### (V) Special details

**Table:**

Geometry. All esds (except the esd in the dihedral angle between two l.s. planes) are estimated using the full covariance matrix. The cell esds are taken into account individually in the estimation of esds in distances, angles and torsion angles; correlations between esds in cell parameters are only used when they are defined by crystal symmetry. An approximate (isotropic) treatment of cell esds is used for estimating esds involving l.s. planes.

### (V) Fractional atomic coordinates and isotropic or equivalent isotropic displacement parameters (Å^2^)

**Table:**

	*x*	*y*	*z*	*U*_iso_*/*U*_eq_	
C1	0.1889 (9)	0.1476 (6)	0.5224 (3)	0.0600 (15)	
C2	0.1527 (15)	0.0459 (7)	0.5969 (4)	0.104 (3)	
C3	0.2121 (8)	−0.1169 (6)	0.5878 (3)	0.0520 (12)	
H3	0.211403	−0.188177	0.636650	0.062*	
C4	0.2731 (8)	−0.1829 (6)	0.5112 (3)	0.0531 (12)	
H4	0.310133	−0.296598	0.507457	0.064*	
C5	0.2788 (17)	−0.0840 (8)	0.4429 (4)	0.126 (4)	
H5	0.303249	−0.124879	0.389331	0.151*	
C6	0.0868 (10)	0.1186 (7)	0.6779 (3)	0.0651 (16)	
H6A	−0.036542	0.210386	0.674726	0.098*	
H6B	0.195698	0.156258	0.691792	0.098*	
H6C	0.057412	0.037620	0.721293	0.098*	
N1	0.2493 (9)	0.0780 (6)	0.4499 (3)	0.0705 (15)	
H1	0.271393	0.138646	0.404236	0.085*	
N2	0.1632 (6)	0.3024 (5)	0.5236 (2)	0.0475 (10)	
H2A	0.185700	0.360039	0.476852	0.057*	
H2B	0.123470	0.349100	0.571202	0.057*	
C7	0.2567 (8)	0.2446 (6)	−0.0094 (3)	0.0501 (12)	
C8	0.3081 (8)	0.1491 (6)	−0.0793 (3)	0.0496 (12)	
C9	0.2983 (7)	−0.0075 (6)	−0.0664 (3)	0.0461 (11)	
H9	0.329701	−0.071870	−0.112816	0.055*	
C10	0.2439 (8)	−0.0740 (7)	0.0125 (4)	0.0584 (14)	
H10	0.242538	−0.183511	0.020582	0.070*	
C11	0.1918 (9)	0.0225 (9)	0.0790 (4)	0.0737 (19)	
H11	0.148649	−0.018341	0.133404	0.088*	
C12	0.3736 (8)	0.2208 (6)	−0.1633 (3)	0.0449 (11)	
H12A	0.483628	0.266937	−0.159640	0.067*	
H12B	0.257460	0.306089	−0.182129	0.067*	
H12C	0.422840	0.136364	−0.203087	0.067*	
N3	0.2029 (7)	0.1758 (6)	0.0657 (3)	0.0631 (12)	
H3A	0.172986	0.234488	0.109125	0.076*	
N4	0.2588 (7)	0.3949 (5)	−0.0157 (3)	0.0602 (12)	
H4A	0.225239	0.450402	0.029029	0.072*	
H4B	0.293896	0.440766	−0.064693	0.072*	
Zn1	0.40814 (8)	0.39971 (6)	0.23744 (3)	0.03752 (16)	
Cl1	0.37981 (18)	0.50225 (14)	0.36231 (7)	0.0428 (3)	
Cl2	0.73745 (17)	0.33366 (14)	0.17781 (8)	0.0471 (3)	
Cl3	0.19082 (18)	0.57548 (14)	0.15631 (7)	0.0460 (3)	
Cl4	0.2998 (2)	0.17366 (15)	0.25763 (7)	0.0508 (3)	
O1	0.1386 (6)	0.5035 (5)	0.6524 (2)	0.0587 (9)	
H1O	0.053464	0.539417	0.697263	0.07 (2)*	
H2O	0.256463	0.514917	0.659163	0.10 (2)*	

### (V) Atomic displacement parameters (Å^2^)

**Table:**

	*U*^11^	*U*^22^	*U*^33^	*U*^12^	*U*^13^	*U*^23^
C1	0.084 (4)	0.041 (3)	0.049 (3)	−0.024 (3)	0.024 (3)	−0.010 (2)
C2	0.198 (9)	0.042 (3)	0.049 (3)	−0.031 (4)	0.044 (4)	−0.006 (3)
C3	0.055 (3)	0.039 (3)	0.060 (3)	−0.015 (2)	−0.002 (2)	0.001 (2)
C4	0.054 (3)	0.040 (3)	0.062 (3)	−0.012 (2)	0.001 (2)	−0.014 (2)
C5	0.244 (12)	0.059 (4)	0.060 (4)	−0.060 (6)	0.062 (6)	−0.029 (3)
C6	0.095 (4)	0.045 (3)	0.044 (3)	−0.018 (3)	0.014 (3)	−0.002 (2)
N1	0.113 (4)	0.052 (3)	0.043 (2)	−0.038 (3)	0.028 (3)	−0.013 (2)
N2	0.063 (3)	0.040 (2)	0.037 (2)	−0.0188 (19)	0.0038 (18)	−0.0008 (16)
C7	0.056 (3)	0.058 (3)	0.035 (2)	−0.014 (2)	−0.005 (2)	−0.008 (2)
C8	0.059 (3)	0.053 (3)	0.039 (3)	−0.019 (2)	−0.007 (2)	−0.003 (2)
C9	0.038 (2)	0.048 (3)	0.054 (3)	−0.011 (2)	−0.014 (2)	−0.002 (2)
C10	0.051 (3)	0.063 (3)	0.067 (4)	−0.027 (3)	−0.018 (3)	0.016 (3)
C11	0.051 (3)	0.112 (6)	0.056 (4)	−0.031 (3)	−0.008 (3)	0.022 (4)
C12	0.055 (3)	0.043 (3)	0.035 (2)	−0.011 (2)	−0.005 (2)	−0.0054 (19)
N3	0.070 (3)	0.086 (4)	0.033 (2)	−0.024 (3)	−0.001 (2)	−0.005 (2)
N4	0.075 (3)	0.056 (3)	0.046 (2)	−0.010 (2)	−0.008 (2)	−0.018 (2)
Zn1	0.0406 (3)	0.0372 (3)	0.0340 (3)	−0.0110 (2)	−0.0026 (2)	−0.0052 (2)
Cl1	0.0489 (6)	0.0443 (6)	0.0345 (5)	−0.0129 (5)	−0.0017 (4)	−0.0090 (4)
Cl2	0.0406 (6)	0.0446 (6)	0.0526 (7)	−0.0090 (5)	0.0021 (5)	−0.0114 (5)
Cl3	0.0487 (6)	0.0458 (6)	0.0403 (6)	−0.0067 (5)	−0.0105 (5)	−0.0042 (5)
Cl4	0.0696 (8)	0.0456 (6)	0.0439 (6)	−0.0282 (6)	−0.0045 (5)	−0.0039 (5)
O1	0.061 (2)	0.070 (2)	0.053 (2)	−0.030 (2)	−0.0003 (18)	−0.0173 (18)

### (V) Geometric parameters (Å, º)

**Table:**

C1—N2	1.306 (6)	C8—C9	1.376 (7)
C1—N1	1.335 (7)	C8—C12	1.494 (6)
C1—C2	1.450 (7)	C9—C10	1.387 (7)
C2—C3	1.368 (7)	C9—H9	0.9500
C2—C6	1.472 (8)	C10—C11	1.380 (9)
C3—C4	1.380 (7)	C10—H10	0.9500
C3—H3	0.9500	C11—N3	1.351 (8)
C4—C5	1.328 (9)	C11—H11	0.9500
C4—H4	0.9500	C12—H12A	0.9800
C5—N1	1.377 (8)	C12—H12B	0.9800
C5—H5	0.9500	C12—H12C	0.9800
C6—H6A	0.9800	N3—H3A	0.8800
C6—H6B	0.9800	N4—H4A	0.8800
C6—H6C	0.9800	N4—H4B	0.8800
N1—H1	0.8800	Zn1—Cl2	2.2536 (13)
N2—H2A	0.8800	Zn1—Cl3	2.2704 (13)
N2—H2B	0.8800	Zn1—Cl1	2.2710 (12)
C7—N4	1.305 (7)	Zn1—Cl4	2.2867 (13)
C7—N3	1.344 (6)	O1—H1O	0.8802
C7—C8	1.420 (7)	O1—H2O	0.8817
			
N2—C1—N1	119.5 (5)	C9—C8—C12	122.6 (4)
N2—C1—C2	122.9 (5)	C7—C8—C12	119.1 (4)
N1—C1—C2	117.6 (5)	C8—C9—C10	121.8 (5)
C3—C2—C1	116.2 (5)	C8—C9—H9	119.1
C3—C2—C6	124.1 (5)	C10—C9—H9	119.1
C1—C2—C6	119.0 (5)	C11—C10—C9	118.5 (5)
C2—C3—C4	123.3 (5)	C11—C10—H10	120.7
C2—C3—H3	118.4	C9—C10—H10	120.7
C4—C3—H3	118.4	N3—C11—C10	119.1 (5)
C5—C4—C3	118.6 (5)	N3—C11—H11	120.5
C5—C4—H4	120.7	C10—C11—H11	120.5
C3—C4—H4	120.7	C8—C12—H12A	109.5
C4—C5—N1	119.8 (6)	C8—C12—H12B	109.5
C4—C5—H5	120.1	H12A—C12—H12B	109.5
N1—C5—H5	120.1	C8—C12—H12C	109.5
C2—C6—H6A	109.5	H12A—C12—H12C	109.5
C2—C6—H6B	109.5	H12B—C12—H12C	109.5
H6A—C6—H6B	109.5	C7—N3—C11	124.4 (5)
C2—C6—H6C	109.5	C7—N3—H3A	117.8
H6A—C6—H6C	109.5	C11—N3—H3A	117.8
H6B—C6—H6C	109.5	C7—N4—H4A	120.0
C1—N1—C5	123.3 (5)	C7—N4—H4B	120.0
C1—N1—H1	118.3	H4A—N4—H4B	120.0
C5—N1—H1	118.3	Cl2—Zn1—Cl3	113.75 (5)
C1—N2—H2A	120.0	Cl2—Zn1—Cl1	109.26 (5)
C1—N2—H2B	120.0	Cl3—Zn1—Cl1	110.27 (5)
H2A—N2—H2B	120.0	Cl2—Zn1—Cl4	109.40 (5)
N4—C7—N3	119.5 (5)	Cl3—Zn1—Cl4	104.48 (5)
N4—C7—C8	122.7 (5)	Cl1—Zn1—Cl4	109.55 (5)
N3—C7—C8	117.8 (5)	H1O—O1—H2O	105.6
C9—C8—C7	118.3 (5)		
			
N2—C1—C2—C3	−171.5 (7)	N4—C7—C8—C9	−179.1 (5)
N1—C1—C2—C3	9.1 (12)	N3—C7—C8—C9	0.6 (8)
N2—C1—C2—C6	−0.9 (13)	N4—C7—C8—C12	1.9 (8)
N1—C1—C2—C6	179.8 (7)	N3—C7—C8—C12	−178.5 (5)
C1—C2—C3—C4	−10.1 (12)	C7—C8—C9—C10	−1.3 (7)
C6—C2—C3—C4	179.9 (7)	C12—C8—C9—C10	177.7 (5)
C2—C3—C4—C5	1.5 (11)	C8—C9—C10—C11	2.2 (8)
C3—C4—C5—N1	8.0 (13)	C9—C10—C11—N3	−2.5 (8)
N2—C1—N1—C5	−179.6 (8)	N4—C7—N3—C11	178.7 (5)
C2—C1—N1—C5	−0.2 (12)	C8—C7—N3—C11	−1.0 (8)
C4—C5—N1—C1	−8.8 (14)	C10—C11—N3—C7	2.0 (9)

### (V) Hydrogen-bond geometry (Å, º)

**Table:**

*D*—H···*A*	*D*—H	H···*A*	*D*···*A*	*D*—H···*A*
N1—H1···Cl4	0.88	2.35	3.134 (5)	148
N2—H2*A*···Cl1	0.88	2.54	3.375 (4)	158
N2—H2*B*···O1	0.88	2.03	2.838 (5)	152
N3—H3*A*···Cl4	0.88	2.66	3.306 (4)	132
N4—H4*A*···Cl3	0.88	2.40	3.268 (4)	170
N4—H4*B*···Cl2^i^	0.88	2.51	3.333 (5)	155
O1—H1*O*···Cl3^ii^	0.88	2.95	3.669 (4)	141
O1—H1*O*···Cl4^ii^	0.88	2.98	3.675 (4)	138
O1—H2*O*···Cl1^iii^	0.88	2.45	3.302 (4)	162
C10—H10···Cl3^iv^	0.95	2.92	3.696 (5)	140

Symmetry codes: (i) −*x*+1, −*y*+1, −*z*; (ii) −*x*, −*y*+1, −*z*+1; (iii) −*x*+1, −*y*+1, −*z*+1; (iv) *x*, *y*−1, *z*.
